# CD147 Promotes Cell Small Extracellular Vesicles Release during Colon Cancer Stem Cells Differentiation and Triggers Cellular Changes in Recipient Cells

**DOI:** 10.3390/cancers12020260

**Published:** 2020-01-21

**Authors:** Donatella Lucchetti, Filomena Colella, Luigi Perelli, Claudio Ricciardi-Tenore, Federica Calapà, Micol E. Fiori, Federica Carbone, Ruggero De Maria, Alessandro Sgambato

**Affiliations:** 1 Fondazione Policlinico Universitario A. Gemelli IRCCS, 00168 Roma, Italy; dnlucchetti@gmail.com (D.L.); ruggero.demaria@unicatt.it (R.D.M.); 2 Institute of General Pathology, Università Cattolica del Sacro Cuore, 20123 Roma, Italy; colella.filomena@gmail.com (F.C.); luigi.perelli19934@gmail.com (L.P.); c.ricciarditenore@hotmail.it (C.R.-T.); federica.calap@gmail.com (F.C.); 3 Department of Oncology and Molecular Medicine, Istituto Superiore di Sanità, 00161 Rome, Italy; fiorimicol@gmail.com; 4 Department of Genitourinary Med Onc, The University of Texas MD Anderson Cancer Center, 1515 Holcombe Boulevard, Houston, TX 77030, USA; fcarbone@mdanderson.org; 5 Centro di Riferimento Oncologico della Basilicata (IRCCS-CROB), 85028 Rionero in Vulture, Italy

**Keywords:** small extracellular vesicles, colorectal cancer stem cells, differentiation, CD147

## Abstract

Cancer cells secrete small extracellular vesicles (sEVs) that are involved in the remodeling of tumor microenvironment (TME) and can promote tumor progression. The role of sEVs and their molecular key players in colon cancer stem cells differentiation are poorly understood. This study aimed to analyze the role and content of sEVs released during the differentiation of colorectal cancer stem cells. Here we show that sEVs secretion during colon cancer stem cells differentiation is partially controlled by CD147, a well-known player involved in colon cancer tumorigenesis. CD147 + sEVs activate a signaling cascade in recipient cells inducing molecular invasive features in colon cancer cells. CD147 knockdown as well as anti-CD147 antibodies impaired sEVs release and downstream effects on recipient cells and blocking multivesicular body maturation prevented sEVs release during the differentiation. Our findings reveal a functional role of CD147 in promoting sEVs release during the differentiation of colon cancer stem cells and in triggering cellular changes in recipient cells.

## 1. Introduction

Despite the advance in diagnosis and treatment of colorectal cancer (CRC) the clinical outcome and the disease-free survival of CRC patients remains poor. The management of CRC patients is a critical challenge mainly due to the development of metastases and therapy resistance [1]. Cancer stem cells (CSC) are a group of cancer cells with the ability to self-renew and differentiate into various non-stem cancer cells, and play a crucial role in cancer progression, metastasis, and resistance to therapy [2,3].

Accumulating evidence suggests that the reciprocal relationships between cancer cells and the surrounding non-neoplastic cells, the so-called tumor microenvironment, is likely pivotal for several aspects of tumor development [4]. On this basis, great attention has been drawn in recent years to factors which could mediate a cross-talk between cancer cells and surrounding microenvironment and several studies have suggested that the intercellular exchange of nucleic acids and proteins within the cancer microenvironment could play pivotal roles in cancer development, progression and formation of the pre-metastatic niche [4,5]. Thus, the tumor microenvironment (TME) appears to have a dominant role in determining the behavior of cancer cells, including cancer stem cells (CSCs), and vice versa. A better understanding of the interactions between cancer cells and the surrounding microenvironment might contribute to our understanding of the process of tumor development and has the potentiality to allow the identification of novel cancer biomarkers and/or therapeutic targets. Extracellular vesicles (EVs) are nano-sized (20–1000 nm) membranous vesicles carrying biologically active material that is transferred to recipient cells as tools of intercellular communication [6]. EVs released by cancer cells or by tumor-stromal cells transmit signals to other (cancer and/or stromal) cells that could affect tumor development and progression [7,8]. The International Society for Extracellular Vesicles grouped the EVs according to their size: Small (sEVs < 200 nm) and medium (mEVs > 200 nm) EVs [9]. Most scientific attention is given to sEVs for their peculiar mechanism of formation [6] and because several evidences have suggested a potential role in tumor progression, metastasis, and recurrence. Differentiation therapy, referring to treating malignant tumors via the induction of CSC differentiation, markedly improved the outcome of acute promyelocytic leukemia but to date, no differentiation drugs have been demonstrated to exert a curative effect on solid tumors [10]. Recently, in vitro and in vivo data linked sEVs to drug-resistance in several cancers but the sEVs molecular key players involved in colon CSC differentiation are poorly understood [11]. CD147 or EMMPRIN (extracellular matrix metalloproteinase inducer) plays an important role in tumor progression, being involved in cancer cell growth, invasion and metastasis, and it is a prognostic factor in CRC [12]. CD147 is a glycoprotein released by tumor cells and transferred to surrounding cells (both cancer cells and TME) in soluble form or by EVs [12]. CD147-sEVs have been identified in ovarian and bladder cancer, and recent evidence suggests that circulating CD147-containing EVs might be used as a biomarker to monitor response to therapy in CRC patients, but no study so far has analyzed the CD147 fate during colorectal cancer stem cell lines (CR-CSCs) differentiation [13,14,15,16]. This study aimed to analyze the role of sEVs in the differentiation process of CR-CSC and to understand whether CD147-sEVs could be involved in differentiation therapy inefficacy.

## 2. Materials and Methods

### 2.1. Cell Culture

HT29 and HCT116 (human colorectal adenocarcinoma) cell lines were used for in vitro studies and were cultured in Dulbecco’s modified Eagle’s medium supplemented with 10% fetal bovine serum (GIBCO, Fisher Scientific UK Ltd., Loughborough, UK), 1% penicillin-streptomycin, and 2 mmol/L l-glutamine at 37 °C in a humid 5% CO_2_ atmosphere. CR-CSC lines (CSC1, CSC2, CSC3, CSC4) were generated were generated in the lab of Prof. De Maria at ISS, as previously described [17] and were cultured in a serum-free medium supplemented with 20 ng/mL epidermal growth factor (EGF) and 10 ng/mL FGF-2 (PeproTech, Rocky Hill, NY, USA) on low-attachment plates. All CR-CSCs were validated for their capability to generate neoplasms faithfully phenocopying the original patient tumor when xenotransplanted into immunocompromised mice. CSC lines were further validated by Short Tandem Repeat DNA fingerprinting. For all CSC lines, profiles were compared against publically available databases and internal database to confirm authenticity. Differentiation was induced by culturing CR-CSCs with medium supplemented with 10% of FBS (Fetal Bovine Serum) in the absence of growth factors for one week and when cells reached the differentiation condition the medium was changed with basal culture medium supplemented only with glutamine and antibiotics followed by EVs collection after 48 h.

### 2.2. Purification and Characterization of sEVs

Conditioned cell culture media enriched in sEVs were centrifuged at 750× *g* for 15 min, and then at 1500× *g* for 5 min. Supernatants were saved and centrifuged at 17,000× *g* for 45 min. Then the pellets composed by microvesicles were washed in phosphate-buffered saline (PBS) by centrifugation at 17,000× *g* for 45 min. Supernatants 0.22 µm filtered were transferred to fresh tubes and centrifuged at 120,000× *g* for sEVs purification. sEVs pellets were resuspended in PBS and used for the treatment of cells or to prepare protein extracts for Western blot analysis. The Bradford assay was used for the quantitative evaluation of sEVs. Size and morphological analysis of sEVs were carried out with dynamic light scattering and transmission electron microscopy, respectively, as previously described [18].

### 2.3. Western Blot Analysis

The cells or sEVs pellet were lysed using lysis buffer (50 mmol/L Tris-HCl pH 7.2, 5 mmol/L MgCl_2_, 50 mmol/L NaCl, 0.25%, 0.1% SDS, and 1% Triton X-100) containing protease inhibitors (2 mmol/L phenyl methyl sulfonyl fluoride, 10 mg/mL aprotinin, and 2 mmol/L Na_3_VO_4_, 100 mmol/L NaF). Differently, for separation of cytoplasmatic, membrane, and nuclear soluble proteins, cells were lysed using Subcellular Protein Fraction kit for Cultured Cells (Thermo Fisher Scientific, Waltham, MA, USA). Protein concentration was assessed using the Bradford method (Bradford protein assay kit II, Bio-Rad, Hercules, CA, USA), with BSA used as a standard. Cell lysates (40 µg) and EVs extracted proteins (10 µg) were resolved by SDS PAGE (Sodium Dodecyl Sulfate PolyAcrylamide Gel Electrophoresis) 10% under reducing or non-reducing conditions and were transferred to PVDF blotting membranes (GE Healthcare, Solingen, Germany) and analyzed using the enhanced chemiluminescence kit for Western blotting detection ((Advansta, WesternBright TM ECL), Bering Drive San Jose, CA, USA)). Primary monoclonal antibodies were used following suppliers’ instructions and included the following: mouse anti-human monoclonal CD9 (dilution, 1:500; Santa Cruz Biotechnology, Inc., Dallas, TX, USA), mouse monoclonal anti-human EMMPRIN (dilution, 1:500; Santa Cruz Biotechnology, Inc.), mouse monoclonal anti-human EMMPRIN (8D6; sc-21746; dilution 1:500; Santa Cruz Biotechnology, Inc.), mouse monoclonal anti-human β-Actin (C4; sc-47778; dilution 1:500; Santa Cruz Biotechnology, Inc.), mouse monoclonal anti-human PARP-1 (N-20; sc-1561; dilution 1:500; Santa Cruz Biotechnology, Inc.), rabbit polyclonal anti-human PROM1 (PAB12663; dilution 1:500 Abnova, Heidelberg, Germany).

### 2.4. RT-qPCR Assays

Total RNA was extracted from cells and corresponding EVs using RiboPure™ RNA Purification Kit (Ambio, Thermo Fisher Scientific UK Ltd.) and cDNA was obtained using the iScript cDNA Synthesis kit (Bio-Rad Laboratories S.r.l., Segrate, Milan, Italy). Each real-time polymerase chain reaction (PCR) was prepared in triplicate and was carried out using SSOADV-univer-SYBR-GREEN (Bio-Rad Laboratories S.r.l., Segrate, Milan, Italy). The sequences of the primers used for PCR were as follows: CD133, EMMPRIN, RAC-1, cdc42, α-sma and b-actin as housekeeping gene (Table 1). Analysis was performed using the CFX96 Touch Real-Time PCR Detection System (Bio-Rad Laboratories S.r.l.), and the acquisition and data processing were performed using the CFX Manager software version 1.6 (Bio-Rad Laboratories S.r.l.).

### 2.5. Immunofluorescence Staining

HCT116 cells were grown in 8-wells culture slides and were fixed with 4% paraformaldehyde for 30 min. Slides, after permeabilization with triton X-100 0.1%/PBS for 10 min, were blocked with 3% normal goat serum/PBS for 30 min at room temperature. They were then incubated with anti-CD147 antibody (mouse monoclonal anti-human, dilution, 1:200; Santa Cruz Biotechnology, Inc., Dallas, TX, USA) overnight at 4 °C, followed by incubation with mouse secondary antibody (dilution 1:500, Alex Fluor 633 conjugated, clone 1H6, Thermo Fisher Scientific UK Ltd.) for 30 min. Afterwards, slides were incubated with fluorescein isothiocyanate–labeled phalloidin for 45 min to counterstained cytoskeleton. Subsequently, the slides were mounted with DAPI (Fluoromount G with DAPI; Electron Microscopy Sciences, Hatfield, PA, USA). Control samples were incubated with secondary antibody only. Slides were examined using an inverted confocal microscope (Nikon A1 MP, Instruments Inc. Melville, NY, USA). Images were taken using ImageJ software version 1.41 (NIH, Bethesda, Rockville, MD, USA). Analyses were performed using the OrientationJ plugins to analyze the coherency [19]. Quantification of nuclear and cytoplasm fluorescence signal was performed using the “Intensity Ratio Nuclei Cytoplasm Tool” plugin of Image J (NIH, Bethesda, MA, USA).

### 2.6. Small Interfering RNA and Transfection

Small interfering (si) RNA (GE Healthcare Dharmacon, Inc. Lafayette, CO, USA) used for transfection was SMARTpool siGENOME Human CD147 (682), a mixture of 4 siRNA provided as a single reagent (GGUCAGAGCUACAUUGA, GAAGUCGUCAGAACACAUC, GUACAAGAUCACUGACUCU, GGACAAGGCCCUCAUGAAC) and siGENOME non-targeting siRNA Pool #1 (UAGCGACUAAACACAUCAA, UAAGGCUAUGAAGAGAUAC, AUGUAUUGGCCUGUAUUAG, AUGAACGUGAAUUGCUCAA). Non-Targeting control siRNA (Dhermacon) was used as a negative control. HT29 and HCT116 cell lines were cultured approximately 25,000 cells/well in a 24 well cell culture dish and were allowed to adhere for 24 h. Transfection was done following the manufacture’s protocol. Briefly, the mixture of 25 nmol/L of siRNA and 0.05–5 μL of DharmaFECT transfection reagent (Dhermacon) were prepared, then were added to antimicotic free complete medium. Cells were incubated at 37° in 5% CO_2_ for 48, 72, and 96 h. Successful inhibition of CD147 expression was routinely confirmed by Western blotting and real-time q-PCR.

### 2.7. ALP Assay

APL activity in CS-CRC cells was detected using a Phosphatase assay kit (A Geno Technology, Inc., St. Louis, MO, USA) according to the manufacturer’s protocol. Briefly, cells were lysed with 1% Triton X-100 for 20 min. Then 50 μL of total cellular lysates were incubated with 50 μL of PA Substrate and 50 μL PA Assay Buffer and the OD values were read at 405 nm for 1 h. Spectrophotometric readings were taken on a SpectraMax1 Plus 384 spectrophotometer (Molecular Devices Corp., Sunnyvale, CA, USA) and were analyzed using the Softmax Pro Software version 4.3 (Molecular Devices Corp.) to obtain the V_max_ values. Relative ALP activity was normalized by the total protein concentration.

### 2.8. Flow Cytometry

2 × 10^5^ CR-CSC cell lines Sa41, Sa47, Re121, and U11 were differentiated in tissue culture plates (six-well) for seven days in CSC medium supplemented with FBS 10% in the absence of growth factors. Following adhesion, indicative of differentiation, CR-CSC were eventually treated with 2 mM NaB, 20 mM NH_4_Cl, 5 µM GW4869, 2 mM NaB + 20 mM NH_4_CL, 2 mM NaB + 5 µM GW4869 and after 48 h tested for the expression of CD44 and CD133 by flow cytometry. Briefly, the cells were collected, washed with PBS and incubated with anti-CD44 (FITC mouse anti-human CD44, BD Pharmigen^TM^, Milano, Italy, 1:20) and anti-CD133 (PE mouse anti-human CD133/2, Miltenyi Biotec, Bologna, Italy, 1:20) antibodies at 4 °C for 1 h. Cells were then washed with PBS, collected by centrifugation and analyzed by Cytoflex S (Beckman Coulter, Indianapolis, IN, USA) and with CytExpert Software (Beckman Coulter Indianapolis). Negative controls were obtained by incubating the cells with PE and FITC Human IgG1 Isotype Control Antibody (Biolegend, San Diego, CA, USA). The assays were repeated at least three times and gave similar results. The data reported are the results of a representative experiment.

### 2.9. Nano-Flow Cytometry

HCT116 cells were cultured into a 12-well dish with media supplemented with 10% of sEVs-depleted FBS (ThermoFisher Scientific, Waltham, MA, USA) and were transfected with siRNA or with non-targeting control siRNA for 96 h. Cell cultured media enriched with EVs were then centrifuged at 750× *g* for 15 min, and then at 1500× *g* for 5 min to remove cells and debris. These supernatants were enriched in both sEVs and mEVs. A part of this supernatant was centrifuged at 17,000× *g* for 45 min and the resulted pellet (mEVs) was suspended in PBS. The remaining supernatant was enriched in sEVs and was saved at −80 °C. 50 μL of EVs, mEVs and sEVs were labelled with 1 μM of Calcein AM (ThermoFisher Scientific). Calcein-AM is converted to green-fluorescent calcein, after acetoxymethyl ester hydrolysis, by intracellular esterases. Damaged vesicles and debris do not express esterase enzymatic activity and do not stain for the dye [20]. EVs counting was analyzed by Cytoflex S instrument (Beckman Coulter, Cassina de´Pecchi, Milano, Italy) equipped with violet laser (405 nm) excitation source. Routinely, time delays between lasers were checked by the standard daily QC startup procedure. The instrument is equipped with peristaltic pump for sample delivery that allows one to record an unlimited sample volume without any dilution effects and without the need of count beads. The sample flow rate used is 10 μL/min and was collected 1,000,000 of events for each sample.

### 2.10. Invasion Assay

Matrigel™ Basement Membrane Matrix (BD) was diluted at the concentration 1 mg/mL and was added to the upper chambers of Transwell^®^ 8.0-mm-sized pores (Transwell, Corning, NY, USA) and incubated at 37 °C overnight. Then the HT29 (1–4 × 10^4^ cell for well) was cultured in upper chamber and 500 μL of medium supplemented with sEV as described or an equal volume of PBS was added to the lower chamber. After 24 h of incubation, the cells were fixed with 4% paraformaldehyde for 10 min. Then, 0.1% crystal violet in 2% ethanol was used to stain cells for 20 min. A cotton swab was used to remove the cells from the up surface of the membranes after washing three times with water. The crystal violet was solubilized with 10% acetic acid and was measured the absorbance at 550 nm with SpectraMax1 Plus 384 spectrophotometer (Molecular Devices Corp, San Jose, CA, USA).

### 2.11. sEVs Internalization

To block CD147 on the sEVs surface, purified sEVs (100 μg) were incubated with anti-CD147 antibody (10 μg/mL) or IgG isotype antibodies (10 μg/mL) in 100 μL PBS for 2 h at 4 °C, and then washed with 30 mL PBS and pelleted by ultracentrifugation to remove the non-bound free antibodies. To analyze sEV uptake by flow cytometry, HCT116 cells were plated into a 24-well plate and were transfected with CD147-siRNA or with CD147 non-targeting control siRNA for 96 h before the treatment with 6 μg of sEVs, sEVs preincubated with anti-CD147 antibodies, sEVs pretreated with N-glycosidase F (15 mU/mL) or sEVs pretreated with Proteinase-K (100 ug/mL) for 2 h at room temperature and for 30 min at 37 °C, respectively. All sEVs were labeled with 1 μM of Calcein AM. After incubation, the cells were collected and washed with PBS solution containing 3% FBS, centrifuged at 1300 rpm for 5 min at 4 °C and analyzed by flow cytometry.

Uptake analysis was performed also by confocal microscopy. Briefly, sEVs were labeled with 1 μM of Calcein-AM or with PE anti-human CD9 Antibody (Clone HI9a, dilution 1:20, Biolegend, London, UK) and administered to cells for 30 min. Imaging was performed by Nikoa A1 Plus (Nikon, Melville, NY, USA) and images were analyzed using the ImageJ software.

### 2.12. Statistical Analysis

Data shown are the means ± standard deviation (SD) of three independent assays. Statistical differences between groups were analyzed using Anova and multiple comparison with Bonferroni test. The difference was considered statistically significant at *p* < 0.05.

## 3. Results

### 3.1. Differentiation of Colon Cancer Stem Cells Increases the Release of Small Extracellular Vesicles (sEVs) and Is Prevented by Its Inhibition

**Figure 1:**
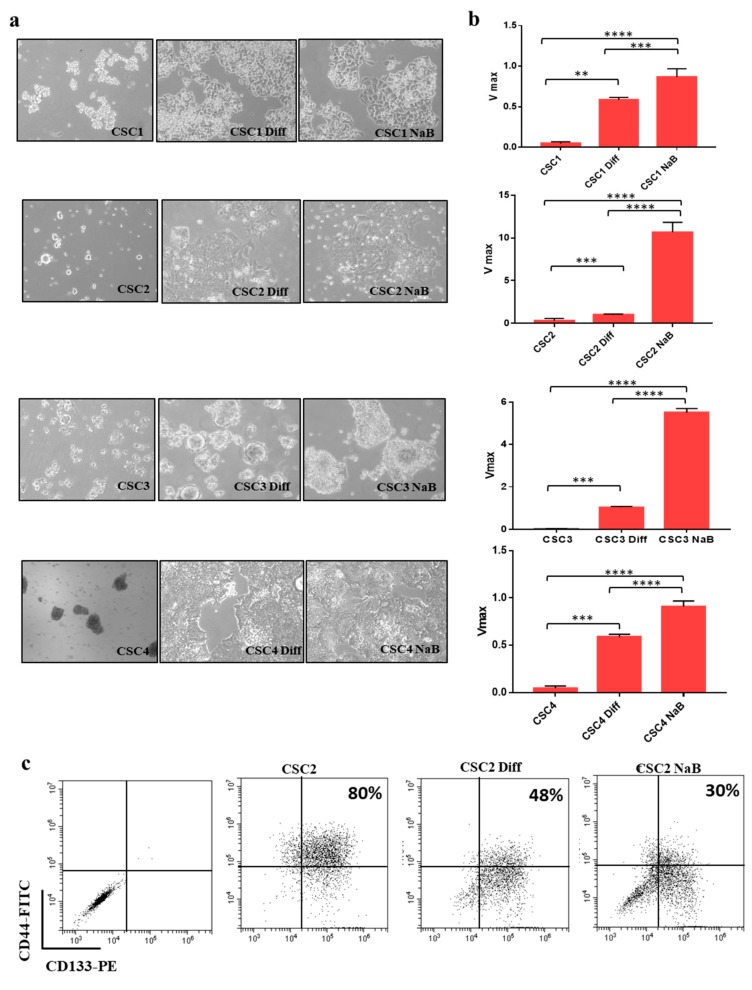
Colorectal cancer stem cell lines (CR-CSCs) can be induced to differentiate. (**a**) CR-CSCs (Sa41, Re121, U11 and Sa47) undergoing differentiation showed morphological changes, magnification= 100×. (**b**) Phosphatase alkaline activity increased in CR-CSC grown in adhesion (Diff) and even more in CR-CSCs treated with NaB (2 mM) thus confirming differentiation. The images of the differentiating CR-CSCs are representative of the whole experiment. (**c**) CR-CSCs markers decreased in differentiated cells. Expression of CD133 and CD44 was assessed by flow cytometry. The double positive cells (CD133^+^/CD44^+^) decreased during the differentiation process (from 80% to 30%). Diff = CR-CSCs were grown with 10% FBS in the absence of growth factors (adhesion condition) for seven days; NaB = CR-CSCs were grown in adhesion condition for seven days and then treated with 2 mM NaB for 48 h. **, *p* ≤ 0.005; ***, *p* ≤ 0.001; ****, *p* ≤ 0.0001.

**Figure 2:**
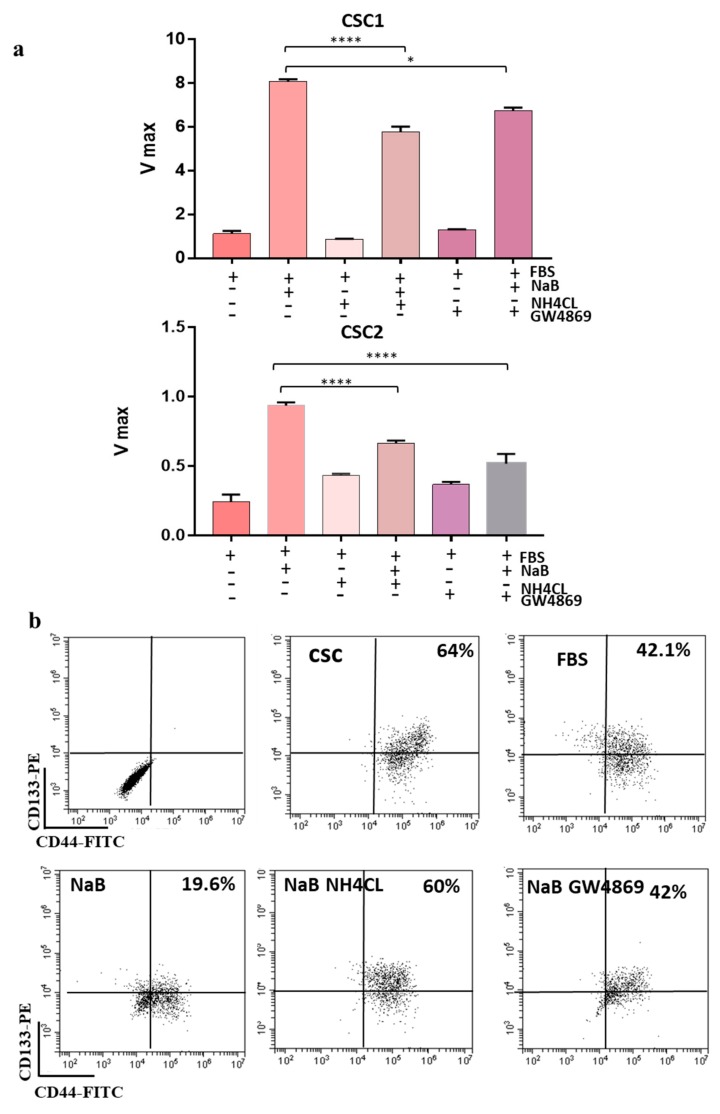
CR-CSCs Differentiation Is Associated With sEVs Release. (**a**) Treatment with 20 mmol/L NH_4_Cl or with 10 μmol/L GW4869, which block multivesicular body maturation, inhibits differentiation of CR-CSCs Sa41 and Re121, as assessed by evaluating ALP activity (Vmax) and (**b**) prevents the reduction of the percentage of CD133/CD44 positive cells (from 19% to 60% and 42% for NH_4_Cl and GW4869, respectively) but similar results were obtained with the other CR-CSC lines tested in this study (Figure S3). CSC = cancer stem cells undifferentiated; FBS = CR-CSCs differentiated with 10% FBS and in absence of growth factors; NaB = CR-CSCs differentiated with 10% FBS, maintained in absence of growth factors and treated after seven days with 2 mM NaB for 48 h. NaB NH_4_Cl = CR-CSCs differentiated with 10% FBS, maintained in absence of growth factors and treated after seven days with 2 mM NaB and 10 mM of NH_4_Cl for 48 h; NaB GW4869 = CR-CSCs differentiated with 10% FBS, maintained in absence of growth factors and treated after seven days with 2mM NaB and 10 μM of GW4869 for 48 h. *, *p* ≤ 0.05; ****, *p* ≤ 0.0001.

Several colorectal cancer stem cell lines (CR-CSCs) isolated from CRC patient carrying different genetic background (CSC1 = KrasG12D/BrafWT; CSC2 = KrasG12V/Braf/WT; CSC3 = KrasWT/BrafV600E; CSC4 = KrasG13D/BrafWT) were used for these studies. Normally, CR-CSC are maintained in a serum-free medium supplemented with 20 ng/mL EGF (Epidermal growth factor) and 10 ng/mL FGF-2 (Fibroblast growth factor-2) and grow in suspension in ultra-low attachment plates. The differentiation of CR-CSCs was induced by two different strategies: Adding FBS 10%, eliminating the growth factors and growing them in adhesion and further adding 2 mM of sodium butyrate (NaB, differentiating agent) after 1 week of FBS. NaB is well known for its ability to induce differentiation of CRC cells [21] but its effects on the differentiation of CR-CSCs have not been explored yet. Differentiation process in CR-CSCs was monitored by assessing phosphatase alkaline activity, as previously reported [18]. Figure 1a shows the morphological changes of CR-CSCs undergoing differentiation. Phosphatase alkaline activity increased in CR-CSC cells exposed to differentiation stimuli compared to undifferentiated counterpart (Figure 1b). Differentiation was also confirmed by the reduction of the population of cells double positive for the CSC markers CD133 and CD44, as assessed by flow cytometry (Figure 1c). We previously reported that NaB-induced differentiation of HT29 and CaCO_2_ colon cancer cells is associated with an increased release of sEV and is prevented by inhibiting sEV release [18]. A similar phenomenon was also observed with CR-CSCs undergoing differentiation by adhesion or by NaB treatment. Indeed, to evaluate the role of sEVs release in the differentiation process of CR-CSCs, we blocked sEVs release by treating cells with NH_4_Cl and/or GW4869, inhibitors of multivesicular body maturation [18]. As expected, differentiation was inhibited when CR-CSCs were treated with NH_4_Cl or GW4869, as assessed by phosphatase alkaline assay (Figure 2a). These data were confirmed testing the double positive cells CD133^+^/CD44^+^: The treatment of CR-CSC with NaB decreased the double positive cells (from 64% to 19%) and this effect was prevented by treatment with NH_4_CL or GW4869 (from 19% to 60% and 42%, respectively) (Figure 2b and Figure S1).

### 3.2. CD147 Has a Key Role in the Biogenesis Of sEVs and Both Its glycoforms Increase during CR-CSCs Differentiation

**Figure 3:**
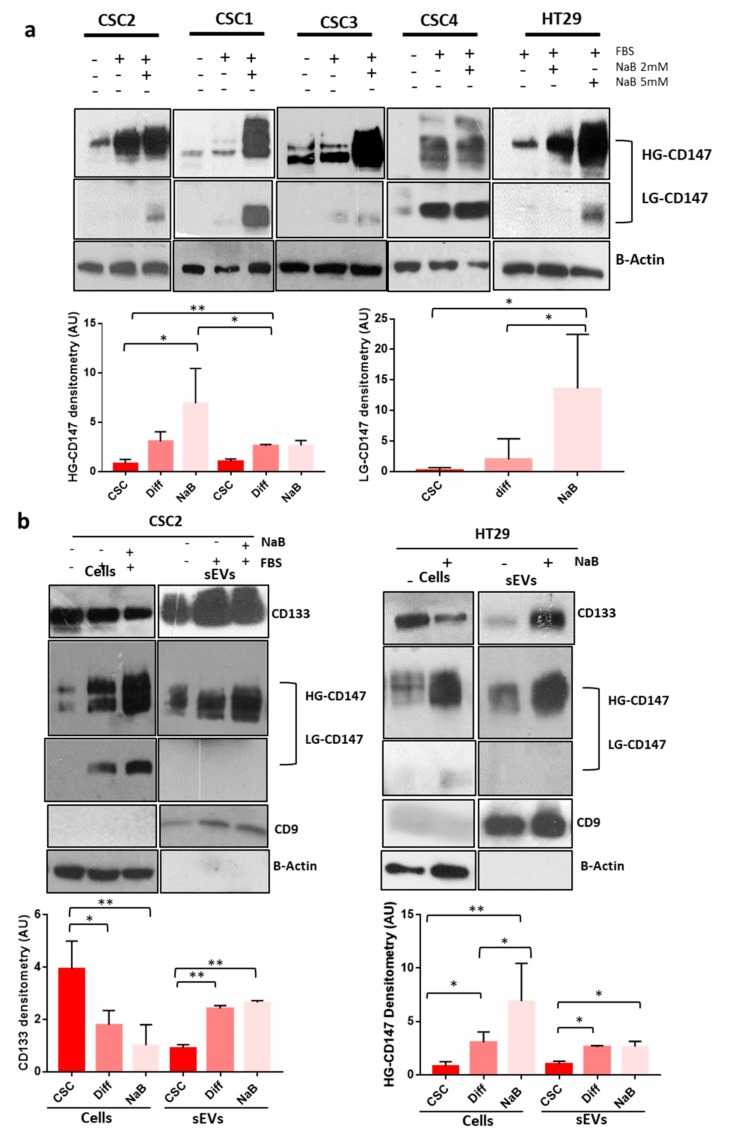
CD147 Expression Increases in CRC (colorectal cancer cells) and CR-CSC (colorectal cancer stem cells) Cell Lines Undergoing Differentiation. (**a**) We evaluated CD147 expression in the HT29 CR cell line and in 4 CR-CSC lines. The differentiation induced an increase of CD147 expression in both glycosylation forms (LG-CD147 and HG-CD147) in all cell lines tested. Bar charts show the densitometry analysis and statistical analysis of CD147 bands in the Re121 CR-CSC and in the HT29 CRC cells lines. (**b**) CD133 and CD147 expression increased in sEVs released by CR-CSCs and HT29 cells lines undergoing differentiation. Bar charts show the densitometry analysis and statistical analysis of both CD133 and CD147 bands in cells and sEVs (small extracellular vesicles) extracts of sEVs of Re121 CR-CSC line undergoing differentiation Data are representative of three independent experiments. Diff = CR-CSCs grow with 10% of FBS (Fetal Bovine Serum) and in absence of growth factors; NaB 2 mM = CR-CSCs grow with 10% of FBS, in absence of growth factors and after seven day (adhesion condition) treated with 2 mM of NaB for 48 h or HT29 treated with 2 mM of NaB for 48 h; NaB 5 Mm = HT29 treated with 5 mM of NaB for 48 h. Analysis of CD9 was used to confirm the correct isolation of sEVs. *, *p* ≤ 0.05; **, *p* ≤ 0.005.

**Figure 4:**
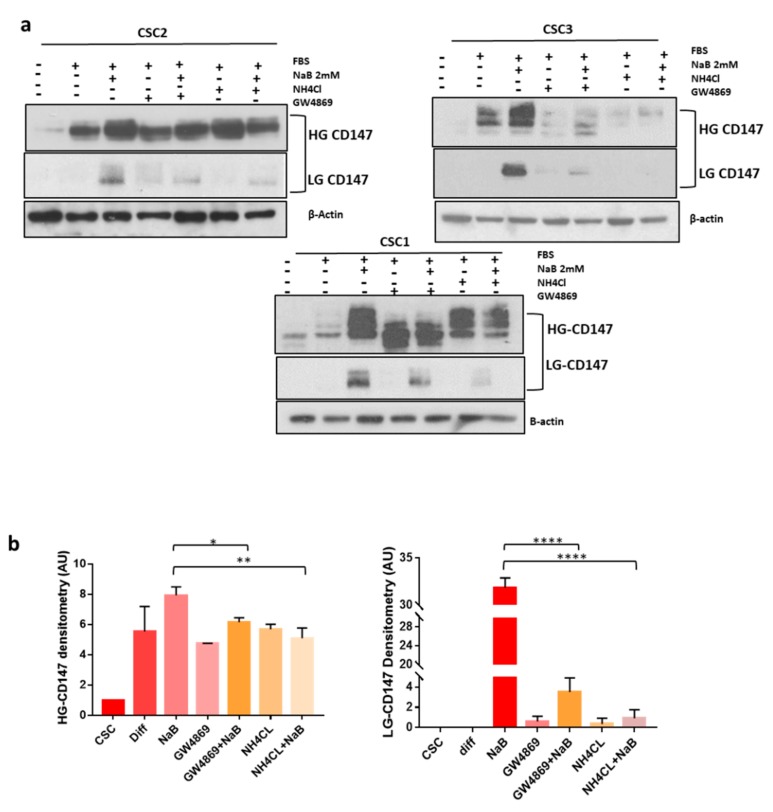
Inhibitors Of sEVs (small extracellular vesicles) Biogenesis Prevent CD147 Upregulation During The Differentiation Of CR-CSC (colorectal cancer stem cell) Lines. (**a**) Simultaneous treatment of CR-CSCs lines induced to differentiate with inhibitors of sEVs release (20 mM NH_4_Cl or 10 µM of GW4869) prevented the increase of cellular CD147 expression and especially of LG-CD147 glycoform. (**b**) Bar charts show the densitometry and statistical analyses of CD147 bands (the data reported are the media of densitometry band of CD147 of three CR-CSCs: Re121, Sa41, U11). Data are representative of three independent experiments. *, *p* ≤ 0.05; **, *p* ≤ 0.005; ****, *p* ≤ 0.0001.

CD147 is a glycoprotein expressed in cells in two glycoforms: Low- (LG) or high- (HG) glycosylated, weighting about 32 and 45–65 kDa, respectively [12]. We evaluated the expression of CD147 in 10 CR-CSC line and in 7-CRC cells lines and found that most of them express both the HG and LG glycoforms of the protein, although at variable levels (Figure S2a). Furthermore, we observed an increase of both CD147 glycosylation forms in CR-CSC lines and in the HT29 cells (Figure 3a). It has been previously shown that CD147 can be released into the microenvironment by extracellular vesicles [22,23,24]. We characterized isolated by TEM, DLS and WB for the expression of CD9 sEVs marker (Figure S3). Moreover, we confirmed the increase of sEVs release during differentiation [18] and their reduction by administration of inhibitors of sEVs release (NH_4_CL or GW4869) (Figure S3). We also evaluated CD147 expression in sEVs and found that only HG-CD147 is carried out from the cells by sEVs shedding and that its expression increased in sEVs released by CR-CSCs during the differentiation process (Figure 3b). Based on these findings, we hypothesized that CD147 might be linked to sEVs release during the differentiation process. To prove this hypothesis, we treated CR-CSCs with inhibitor of sEVs release upon differentiation and analyzed CD147 expression. As expected, inhibition of sEV release prevented the increase of both cellular CD147 glycoforms during the differentiation process (Figure 4a,b). To further evaluate whether the decrease in CD147 expression induced by inhibitors of sEVs release was the result of changes in the expression level of the corresponding mRNA, RT-PCR analysis was used to analyze the levels of CD147 mRNA in CRC cells and in sEVs counterpart. We observed significant changes in the level of CD147 mRNA in HT29 cells treated with inhibitors of sEVs release upon differentiation as well as in the secreted sEVs (Figure S2b).

**Figure 5:**
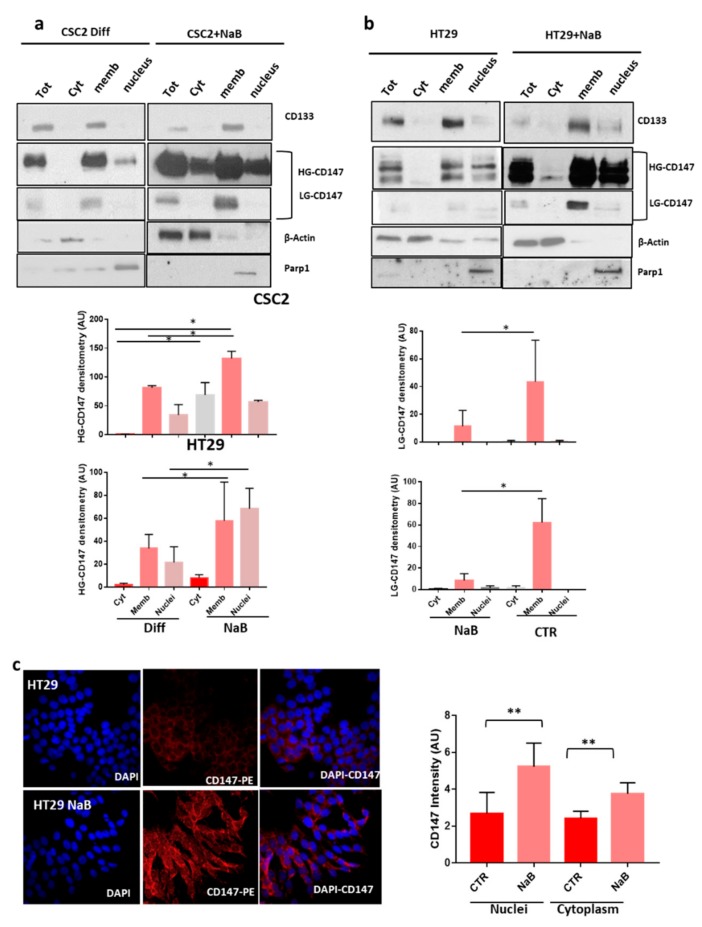
Characterization of CD147 Subcellular Localization In CR-CSC (colorectal cancer stem cell) and HT29 Cell Lines. (**a**,**b**) CD147 expression was analyzed in total cell extracts (Tot) and in the cytoplasmic (Cyt), membranous (Memb) and nuclear (nuclei) fractions of the indicated cell lines. Efficacy of subcellular fractionation is indicated by cytoplasmic marker protein actin, membrane marker protein CD133, and nuclear marker protein Parp1. HG-CD147 was mainly expressed in membrane and nuclear fractions and increased in differentiated cells. Expression of LG-CD147 was confined only to membrane in both cancer cell line and did not change upon differentiation. Bar charts show the densitometry and statistical analyses of CD147 bands. (**c**) Immunofluorescence staining of CD147 in HT29 cancer cell lines treated with NaB compared to untreated cells. DAPI (4′,6-diamidino-2-phenylindole, blue) stains the nuclei; anti-CD147 antibody is labelled with phycobiliprotein (PE) (red), magnification 600×. Bar charts showing the intensity of CD147 staining in nuclei and cytoplasm. Data are representative of three independent experiments. *, *p* ≤ 0.05; **, *p* ≤ 0.005.

To track the localization of CD147 in cellular compartments during the differentiation process, we collected subcellular fractions and analyzed them by western blot. Interestingly, while LG-CD147 was detected only on membranes, the HG-CD147 isoform is present also in the nuclear and cytoplasmic fractions and its expression increased following differentiation in both HT29 and cancer stem cells (Figure 5a,b). These data were confirmed by immunofluorescence intensity analysis of CD147 nuclei/cytoplasm expression in HT29 cancer cells (Figure 5c). Moreover, we analyzed the morphology of CSC4 and HT29 cancer cells after the administration of sEVs inhibitors and analyzed the cellular localization of CD133 and CD147 upon blocking of the sEV release (Figure S4a,b). The cellular localization of CD133 and CD147 did not change blocking sEV release with GW4869. However, this treatment induced an increased expression of CD133 in the membrane compartment, as previously reported (Figure S4b) [18].

**Figure 6:**
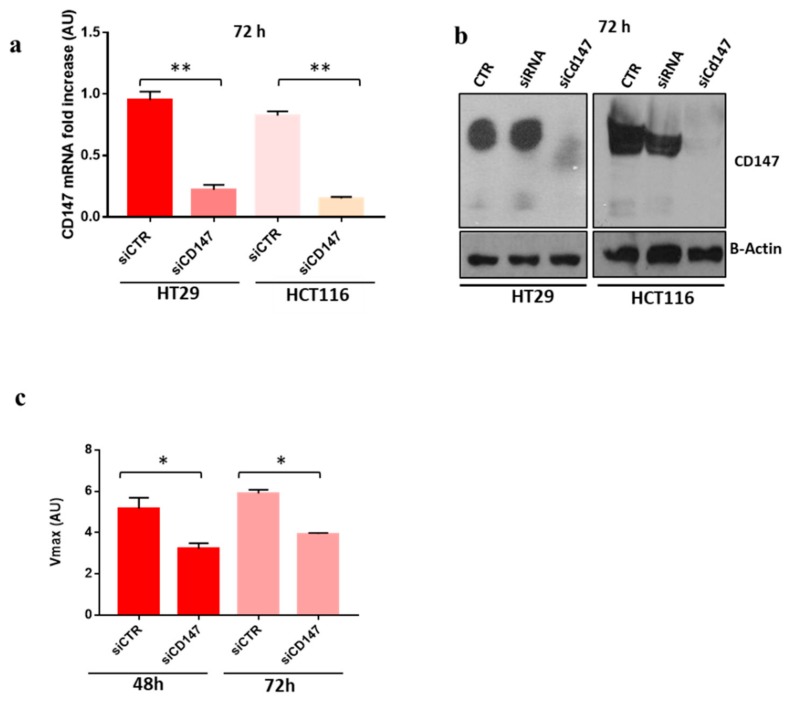
Knock-Down of CD147 Gene By RNA Interference. (**a**,**b**) The knock down of CD147 gene was confirmed by RT-PCR (left) and western blot (right). (**c**) The rate of cell differentiation, assessed by phosphatase alkaline assay, was inhibited in HT29 cells transfected with siRNA for CD147. Data are representative of three independent experiments. siCTR = cells transfected with non-targeting control siRNA; siCD147 = cells transfected with siRNA for specific CD147. *, *p* ≤ 0.05; **, *p* ≤ 0.005.

**Figure 7:**
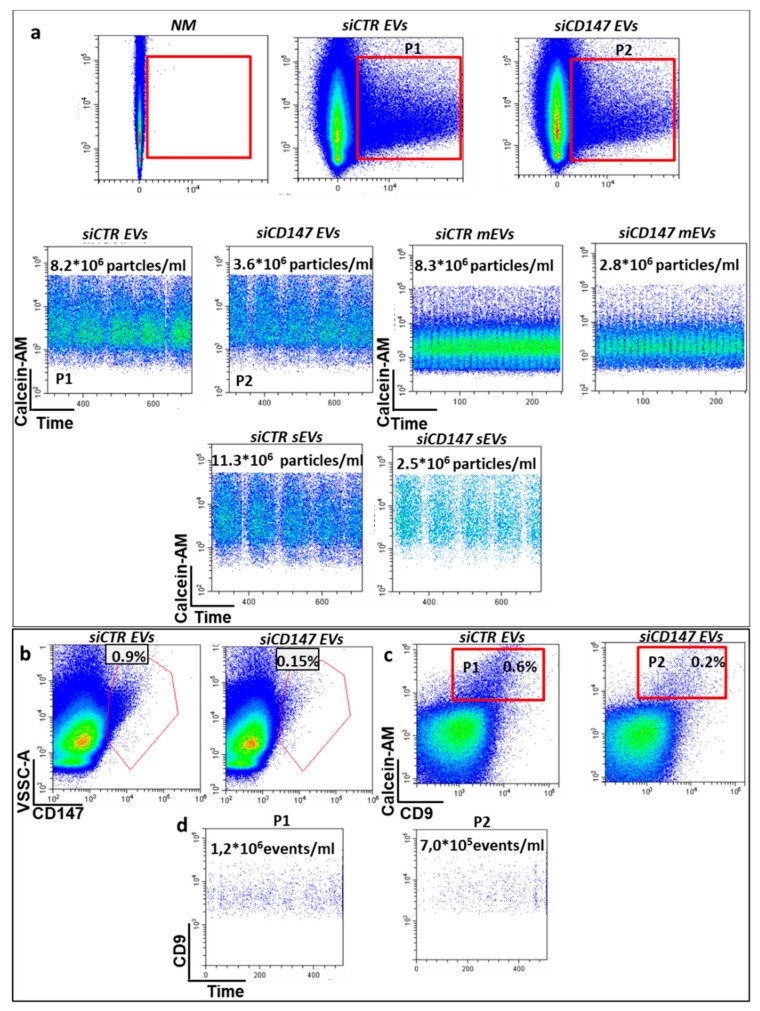
Nano-flow cytometry analysis of extracellular vesicles (EVs). (**a**) The amount of EVs released by colon cancer cells was assessed by Calcein-AM staining (1 µM). To count the events (EVs), we maintained fixed the acquisition time (600 s for sample) thus demonstrating that CD147 downregulation (siCD147) induces a decrease in all fractions of EV although mostly in smaller ones; (**b**) Decrease of CD147-EVs in CD147 knockdown cells (siCD147 EVs); (**c**) EVs staining with CD9 and (**d**) counting to confirm the decrease of EVs released. The images are representative of three independent experiments. NM = EVs, non-stained small extracellular vesicles (sEVs) or medium extracellular vesicles (mEVs); siCTR sEVS = EVs, sEVs or mEVs released by HCT116 transfected with non-targeting control siRNA; siCD147 = EVs, sEVs or mEVs released by HCT116 transfected with specific anti-CD147 siRNA. P1 and P2 = gating strategy for EVs counting.

To confirm the hypothesis that CD147 might play an important role in sEVs biogenesis we used specific anti-CD147 small interfering RNA (siRNA) to inhibit its expression in HT29 and HCT116 cancer cells. CD147 knock down was confirmed by real time PCR and western blot analysis at 48 h, 72 h, and 96 h (Figure 6a,b and data not shown). Upon CD147 knock-down the proliferation rate of HT29 and HCT116 cancer cells was decreased to approximately 20%, as previously reported by Chen (data not shown) [25]. Of note, treatment with siCD147 impairs differentiation of CRC cells, as shown by phosphatase alkaline activity assay, thus suggesting an active role of CD147 in the differentiation process (Figure 6c). To confirm the involvement of CD147 in sEVs release, we quantified EVs release by nanoflow cytometry following Calcein-AM staining. CD147 knock-down in HT29 cancer cells induced a two-fold decrease of whole Calcein-AM positive extracellular vesicles (20–1000 nm in size), three-fold decrease of vesicles with size from 150 to 200 nm (mEVs) and four-fold decrease of sEVs (<150 nm in size) compared to mock-transfected cells (Figure 7). We confirmed the reduced expression of CD147 in sEVS upon CD147 knockdown by both Nano-flow cytometry (Figure 7b) and western blotting (Figure S5b). Moreover, we analyzed the staining of EVs with CD9 (a well know marker of sEVs) and confirmed the decrease of CD9 positive EVs by nano-flow cytometry (Figure 7c,d). To further confirm this finding, given the small size of sEVs, we incubated the conditioned cell culture medium of HT29 cells or the same medium depleted of large vesicles, stained with Calcein-AM, with 4 µm sulfate-beads able to bind sEVs and evaluated them by flow cytometry confirming the reduction of sEVs release in CD147 knock-down cells compared to mock-transfected cells (Figure S5a). Similar results were obtained with HT29 cells (data not shown). We confirmed the decrease of sEVs amount in CD147-knockdown cancer cells using the Bradford assay (Figure S5c).

### 3.3. CD147 Mediates sEV-Effects in Recipient Cells

**Figure 8:**
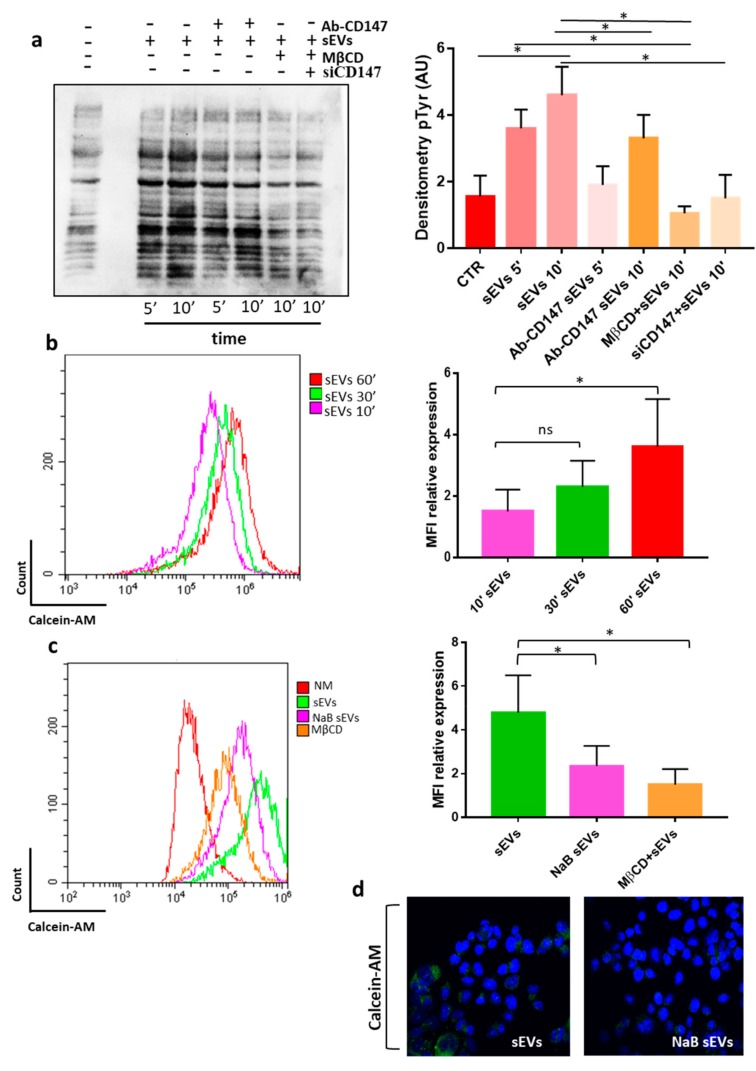
CR-CSC (colorectal cancer stem cell) derived sEVs (small extracellular vesicles) increase the phosphorylation state of intracellular proteins in recipient cells through a CD147-dependent mechanism. (**a**) Western blot analysis of phosphorylated tyrosines in HCT116 cells incubated for 5 (5’) or 10 (10′) min with 10 µg/mL of Re121-sEVs. Ab-CD147 = Re121-sEVs were pre-treated with anti-CD147 antibodies; mβCD = HCT116 were pretreated for 30 min with (1 mM) methyl-β-ciclodextrin, an inhibitor of EVs uptake; siCD147 = Cd147-knock down HCT116 cells; data representative of three independent experiments are shown by bar charts on the right; (**b**) cellular uptake of Calcein-AM labelled sEVs by HCT116 cells at different time-points (10′, 30′, 60′); data representative of three independent experiments are shown by bar charts on the right; (**c**) sEVs released by undifferentiated CR-CSC cells are more efficiently internalized by HCT116 cancer cells compared to sEVs released by NaB differentiated Re121cells (NaB sEVs). The treatment of HCT116 recipient cells with methyl-β-cyclodextrin (mβCD) strongly prevented the uptake of sEVs; data representative of three independent experiments are shown by bar charts on the right; Graph bars (**b**,**c**) indicate the MFI (medium fluorescence intensity) of sEVs internalized and analyzed by flow cytometry. The images are representative of three independent experiments. (**d**) Immunofluorescence staining of HCT116 recipient cells treated for 1 h with sEVs stained with Calcein-AM, Magnification 600×. *, *p* < 0.05.

Based on the above findings, we set out to explore potential signaling mechanisms involved in the sEVs-induced effects on recipient cells. We performed an immunoblot analysis of phosphorylated tyrosine (p-Tyr) to detect changes in the phosphorylation state of intracellular proteins induced by the incubation with sEVs. We observed an overall increase in the level of tyrosine phosphorylation in HCT116 cells treated with CR-CSC-derived sEVs, suggesting that they might activate intracellular signaling pathways by ligand-receptor interactions or by uptake in recipient cells (Figure 8a). To analyze the role of CD147 in such effects on intracellular signaling pathway we pre-treated sEVs with anti-CD147 antibodies and observed that this treatment almost completely abolished them in HCT116 cells (Figure 8a), thus suggesting an important role of sEVs CD147 in sEVs communication with recipient cells. To evaluate whether cellular CD147 is also involved in sEVs effect on recipient cells, we repeated the experiment using CD147-knock down HCT116 cells. As shown in Figure 8a, we found that CD147-knock down in recipient cells can also prevent the increase in the level of tyrosine phosphorylation thus suggesting that the activation of tyrosine cascade in recipient cancer cells might be mediated by a CD147 homophilic interaction between cellular and sEVS CD147 molecules (Figure 8a). Indeed, it was reported that CD147 can self-associate in a cis or trans fashion (homophilic interaction) or cluster with other proteins (heterophilic interaction) [26].

**Figure 9:**
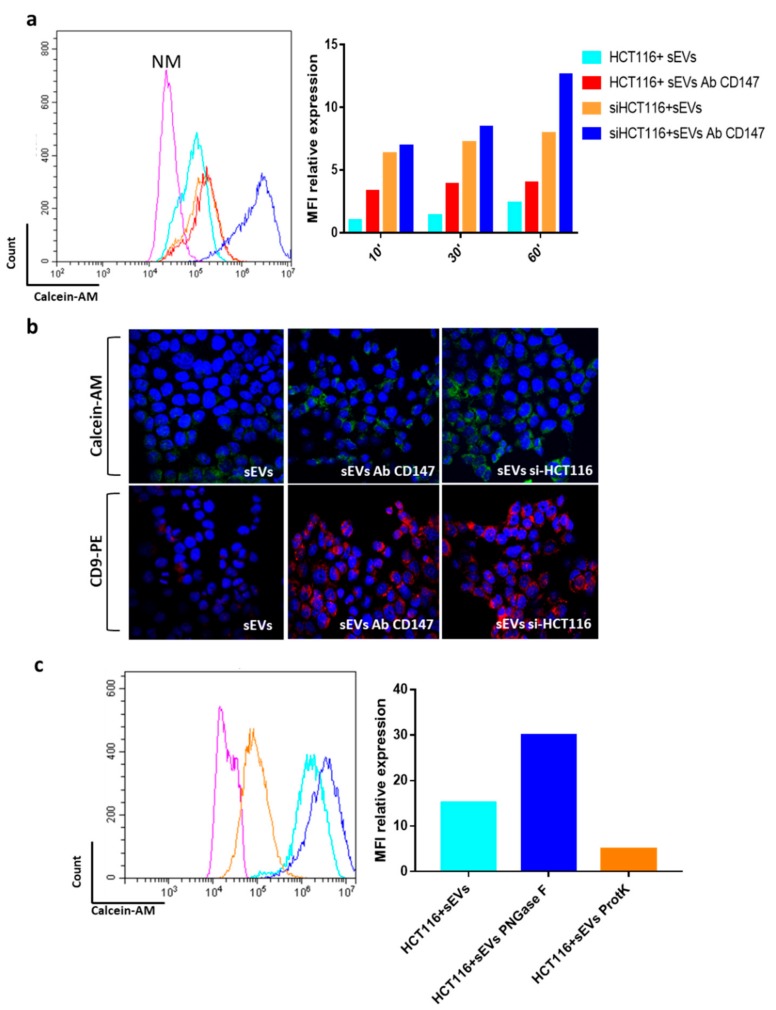
Blocking CD147 ON sEVs increases their cellular internalization. (**a**) Analysis of sEVs (small extracellular vesicles) uptake in recipient HCT116 and in CD147 knockdown HCT116 cells (siHCT116) compared to the uptake of sEVs blocked with CD147 antibody (sEVs CD147); data representative of three independent experiments are shown by bar charts on the right; (**b**) qualitative confirmation of Calcein-AM or anti-CD9 antibodies stained sEVs uptake in control HCT116 and in CD147 knockdown HCT116 cells (si-HCT116) by confocal microscopy. DAPI (blue) = staining nuclei; Calcein-AM = green; CD9-PE = red. (**c**) Same experiment as described in (**a**) using sEVs pretreated with proteinase K (ProtK) or N-Glycosidase F (PNGase F). Representative examples of staining after 1hours of incubation with CR-CSC–released sEVs. The images are representative of three independent experiments. NM = HCT116 treated for 1 h with Re121-sEVs not-stained with 1 μM Calcein-AM; HCT116 sEVs = HCT116 recipient cells treated for 1 h with sEVs stained with 1 μM Calcein-AM; siHCT116 + sEVs = HCT116 recipient cells transfected with non-targeting control and treated for 1 h with Re121 sEVs stained with 1 μM Calcein-AM; HCT116 + sEVs Ab CD147 = HCT116 cells treated with Re121 sEVs CD147 blocked with antibody. Magnification= 600×.

An important issue in the extracellular vesicles field is whether and how they are eventually internalized by recipient cells [27]. Proteins expressed on the membranes of sEVs might be important in mediating their targeting and internalization by recipient cells. We aimed to evaluate whether the above effects could be associated with an increased uptake of sEVs. Treatment with the endocytosis inhibitor methyl-β-ciclodextrin (mβCD), which blocks sEVs uptake in several cell systems, prevented the phosphorylation of p-Tyr in recipient cells thus further confirming the importance of sEVs uptake in the activation of intracellular signaling (Figure 8a). For this reason, we used flow cytometry analysis to evaluate the uptake of calcein-AM stained sEVs at different time points (10′–30′–60′) in HT29 and HCT116 cancer cells (Figure 8b and data not shown) and compared the internalization of sEVs released by differentiated cells (NaB sEVs) with the internalization of sEVs isolated from undifferentiated CRC-CSCs. We found that EVs are incorporated in a time dependent manner by recipient cells and, surprisingly, that sEVs released by differentiated cells are incorporated with a reduced efficiency than sEVs isolated from undifferentiated cells (Figure 8b,c). To further strengthen this finding, we also employed confocal microscopy which confirmed a reduced internalization of NaB sEvs compared to control sEVs (Figure 8d). The experiments described above demonstrated that sEVs isolated from non-differentiated CR-CSCs have a lower CD147 expression than those isolated from differentiated CR-CSC (Figure 3a). We therefore aimed to evaluate whether CD147 was involved in modulating the internalization of sEVs. Therefore, we incubated sEVs with anti-CD147 antibodies (CD147 Ab) before incubating them with recipient cells or we knocked down the CD147 gene in recipient cells and checked sEVs uptake. The blockage of CD147 binding sites on sEVs surface by anti-CD147 antibodies as well as silencing of CD147 in recipient cells increased sEVs uptake in recipient cells (Figure 9a). These effects were further evident when CD147-knock down cells were exposed to anti-CD147 antibodies pretreated sEVs (Figure 9a).

To verify these data, we assessed by confocal microscopy the uptake in recipient cells of sEVs labelled with Calcein-AM or with PE-conjugated anti-CD9 (putative marker of sEVs) antibodies. We observed and confirmed an increased uptake when sEVs were pre-treated with anti-CD147 antibodies as well as when recipient cells were treated with specific anti-CD147 siRNA (Figure 9b). Recently, it has been shown that N-glycans are key players in the tuning of sEVs uptake [28]. CD147 is an N-glycosylated protein so we hypothesized that this post-translational modification may be involved in sEVs cellular uptake. Indeed, we found that the treatment of sEVs with N-glycosidase F increased their internalization in HCT116 recipient cells (Figure 9c). On the other hand, as expected, treatment of sEVs with Proteinase-k prevented their uptake in recipient cells (Figure 9c).

### 3.4. CD147-sEVs Enhanced the Tumorigenic Potential of Cancer Cells

**Figure 10:**
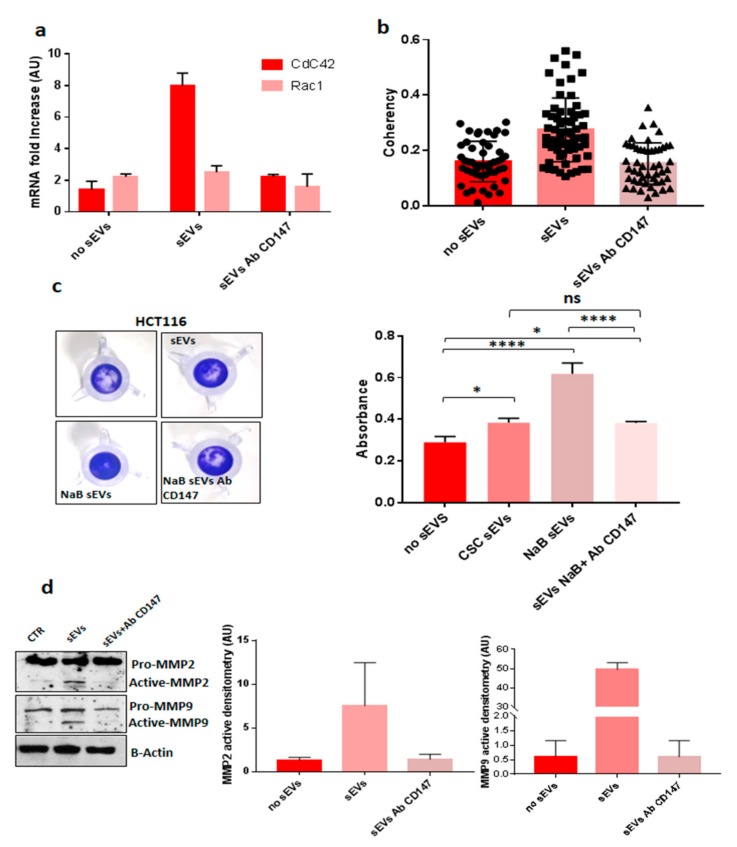
CD147 carried by sEVs (small extracellular vesicles) affects CDC42 expression, actin organization and invasive capacity of recipient cells. (**a**) CR-CSC-sEVs administrated to HCT116 induced an increase of CdC42 expression compared to untreated cells; pre-treatment of sEVs with CD147 antibody prevented the activation of CdC42 expression. (**b**) Analysis of actin cytoskeleton of HCT116 treated with sEVs compared to untreated cells; pre-treatment of sEVs with anti-CD147 antibodies prevented their effect on actin organization; (**c**) invasive assay of HCT116 treated with sEVs: CD147-sEVs increased the invasive potential of recipient cells; (**d**) blocking CD147 on sEVs surface prevented the activation of MMP2-MMP-9 expression. Densitometry of activated MMP2 and MMP9 Data shown are representative of three independent experiments. No sEVs = HCT116 sEVs untreated; sEVs = HCT116 cells treated with Re121-sEVs; sEVs Ab Cd147 = HCT116 cells treated with sEVs CD147 blocked with antibody; NaB sEVs = sEVs released by Re121 differentiated with 10% FBS, maintained in absence of growth factors and treated after seven days with 2 mM NaB; CSC sEVs = HCT116 treated with sEVs released by Re121 CR-CSC; sEVs + Ab Cd147 = HCT116 treated with sEVs released by Re121 CR-CSC and CD147 blocked with antibody. *, *p* ≤ 0.05; **; ****, *p* ≤ 0.0001.

Recent evidence has demonstrated that CD147 is involved in the invasion program of cancer cells through the activation of CdC42, a RhoGTPase involved in cell polarity, migration and actin cytoskeleton organization [29]. We showed that sEVs isolated from Re121 CR-CSC carry CD147 protein (Figure 3a). Thus, it was of interest to analyze whether they are able to modulate CdC42 in recipient cells. We found that sEVs increase the expression of Cdc42, but not of Rac1 (another RhoGTPase), in HCT116 recipient cells and that this effect was prevented by blocking CD147 on the surface of sEVs (Figure 10a). Moreover, we used confocal microscopy to study the actin cytoskeleton organization and to evaluate the actin fiber coherency. The coherency is calculated from the structure tensor of each pixel in the images and is bounded between 0 (isotropic areas) and 1 (highly oriented structures). Greater actin organization is related to increased cell motility [30]. We found that sEVs isolated from Re121 CR-CSC induced an important reorganization of actin filaments in recipient HCT116 cells associated with an increased coherency, indicating a higher organization of cytoskeleton, compared to untreated HCT116 cancer cells. Pre-treatment of sEVs with anti-CD147 antibodies decreased these effects on actin cytoskeleton (Figure 10b).

As mentioned before, CD147 been reported to enhance the invasive potential of cancer cells in part due to overexpression of MMP-2 and MMP-9 (Metalloproteinase 2 and 9) [12]. We found that sEVs released by differentiated CSC3, which are loaded with an increased amount of CD147, increased the invasive potential of HCT116 recipient cells compared to untreated recipient cells and those treated with sEVs released by undifferentiated CR-CSC (Figure 10c). Pre-treatment of sEVs with anti-CD147 antibodies prevented these effects as well as the overexpression of MMP-2 and MMP-9 in HCT116 recipient cells thus demonstrating the central role of CD147 in this process (Figure 10c,d).

## 4. Discussion

CSCs represent a small subset of cells within the tumor bulk that sustain its growth and resist to cancer therapy being involved in tumor recurrence and metastasis which mainly affect prognosis and clinical outcome [2]. How CSC can exert these effects it is still under debate, but several studies showed that sEVs could be key player in cancer metastasization [31,32]. The potential involvement of tumor-derived sEVs in the process of CR-CSC differentiation is also poorly understood and may be a key point to explore given that the differentiation of CR-CSC could be an attractive therapeutic strategy. Dai et al. demonstrated that subpopulations of tumor cell lines that constitutively express high levels of cell-surface CD147 exhibit cancer stem-like cell features and Yoshioka and colleagues confirmed a high level of CD147 expression in EVs isolated from CRC cell lines [13,33]. Despite the interest in CD147, its involvement in the differentiation of CR-CSC and in the effects of sEV on recipient cells has not been fully elucidated. In this study, we demonstrate that CD147 is expressed in all CR-CSC lines analyzed and that it is present in two glycoforms: LG-CD147 and HG-CD147 (Figure S2). Surprisingly, the differentiation of CR-CSC induced a significant increase in the expression of both CD147 glycoforms (Figure 3a). Mohamed et al. showed similar results: during the differentiation of myotubes they noted a change in the molecular forms of CD147 with gradually increase of HG- and LG-CD147 [34]. In particular, we showed that LG-CD147 is expressed only in the membrane compartment and its expression increased in CR-CSCs undergoing to differentiation while HG-CD147 is up regulated in all cellular compartments including nucleus (Figure 5). In this study, we confirmed in CR-CSCs our results previously obtained in immortalized cancer cell lines (HT29 and CaCO_2_) [17]: A relationship exists between differentiation and the increase of sEVs release which sustains the hypothesis that cells adopt sEVs to get rid of molecules, such as CSC markers CD133 (Figure 3b), and to send specific signals to surrounding cells. During the differentiation of CR-CSCs or CRC cell lines the expression of cellular and sEV CD147 increased and we have shown that the differentiation of CR-CSCs is related to active release of sEVs mediated by CD147. These results may indicate that the HG-CD147 could translocate into the nucleus and activate different cellular pathways including those related to the release and biogenesis of sEV. Data obtained by Wu et al. support our hypothesis showing that CD147 contains the classical nuclear leading sequence destined for transport into the nucleus and that this translocation might have a direct role in gene regulation [35].

It is well known that EVs can activate signaling pathways when administered to recipient cells and are capable of inducing pathways involved in cancer initiation, progression, and metastasis [5]. Our results showed that CD147 enriched-sEVs are able to activate phosphorylation of tyrosines in recipient cells and that this phenomenon is prevented when CD147 ligand property is blocked on EVs or its expression is suppressed in recipient cells (Figure 8a). The sEVs once attached to a target cell can activate intracellular signaling pathways via receptor–ligand interaction or can be internalized by endocytosis or even fuse with the membrane of target cells to deliver their content into its cytosol, thus changing the behavior of the recipient cells, as described by Tkach et al. [36]. We think that the activation of tyrosine cascade in recipient cancer cells might be mediated by a CD147 homophilic interaction between cellular and sEVs CD147 molecules (Figure 8a). Indeed, it was reported that CD147 can self-associate in a cis or trans fashion (homophilic interaction) or cluster with other proteins (heterophilic interaction) [26]. Our data showed that CD147 carried by sEVs induce activation of signaling pathways when it interacts with other CD147 molecules expressed on the surface of recipient cells because the uptake of sEVs increased when CD147 is blocked by antibodies or in CD147-suppressed recipient cells but, in the same time, the activation of intracellular pathways, as well as the effects on invasive and motility signaling pathways, was prevented (Figure 8a and Figure 9). These results confirmed the involvement of CD147 in mediating sEVs effects on recipient cells. Accumulating evidences suggest the importance of glycosylation in EVs field [37]. Liang et al. provided strong evidence that N-linked glycosylation directs glycoprotein sorting into sEVs [38], while Royo et al. reported that the modification of the glycosylated complexes on the EV surface could alter the biodistribution of these sEVs in mice [39]. More recently, Williams and colleagues showed that N-glycosylation is related to decrease of sEVs cellular uptake [28]. We confirmed these findings by treating sEVs with N-glycosidase F which promoted cellular uptake of sEVs in recipient cells (Figure 9c).

This observation suggests that N-glycosylation of CD147 could play a key role in mediating the effects induced by sEVs in recipient cancer cells, but surely, other studies are needed to confirm this hypothesis.

It has been reported that both LG- and HG-CD147 contribute to MMP activity in cancer cells, but the efficiency of this induction may be more pronounced referring to HG-CD147 glycoforms [40].

We showed that only the HG-CD147 is sorted in sEVs; similar data have been reported in a study by Menck et al. on breast tumor-derived EVs [41]. Moreover, our data showed that HG-CD147 expressed in sEVs induced the upregulation of MMP-2 and MMP-9 genes expression in recipient cells. Braundmeier et al., also suggested that CD147-microvesicles secreted by human uterine epithelial cells stimulated MMP2 expression in human uterine fibroblast cells [24].

Overall, our data could highlight one of the possible mechanisms by which CR-CSC can resist to differentiation therapy in solid tumors: CD147-sEVs released during differentiation of CR-CSC affect cancer recipient cells tumorigenic property. However, despite the promising results of this study, further investigations are needed to clarify the role of sEVs during the differentiation of CSCs. Moreover, it will be important to analyze the role of CD147-sEVs released by differentiating CR-CSC in mediating sEV effects on other cells of tumor microenvironment, such as cancer associated fibroblasts or immune cells, which could allow to highlight other mechanisms used by CSCs to transmit signals contributing to resistance of cancer cells to differentiation therapies.

## 5. Conclusions

EVs are important mediators of cell communication in tumor microenvironment contributing to tumor progression and metastasis. For this reason, we wondered whether the stress induced by differentiation in colorectal cancer stem cells might modulate their production and secretion of EVs which could alter cell communication within tumor microenvironment. Based on our results, a potential therapeutic strategy could be blocking the effects of CD147-EVs in the tumor microenvironment through the administration of antibodies directed against CD147, together with the induction of tumor differentiation. In this way, it could be possible to obtain the reduction of the tumorigenic potential of tumor cells through the administration of differentiation agents and, on the other hand, it could be neutralized the effects induced by the sEVs released during the differentiation of CR-CSC (which could be the cause of the unsatisfactory results of the differentiation therapeutic strategy in solid tumors).

## Figures and Tables

**Table 1:** Sequence of primer used for RT-qPCR.

Gene	Primer Sequence
β-ACTIN	Forward: 5′-TCTACAATGAGCTGCGTGTGG-3′
	Reverse: 5′-CTGGATAGCAACGTACATGGC-3′
EMMPRIN	Forward: 5′-CAGAGTGAAGGCTGTGAAGTCG-3′
	Reverse: 5′-GCAGTGTGGTCCTCCACTCTCAA-3′
CDC42	Forward: 5′-GCAGGGCAAGAGGATTATGAC-3′
	Reverse: 5′-CAGTGGTGAGTTATCTCAGGC-3′
RAC1	Forward: 5′-AAGTGGTATCCTGAGGTGCG-3′
	Reverse: 5′-TAGACCCTGCGGATAGGTGA-3′

## Supplementary Materials

The following are available online at https://www.mdpi.com/2072-6694/12/2/260/s1, Figure S1: sEVs release is important for the differentiation of CR-CSCs, Figure S2: Evaluation of CD147 expression in CR-CSCs and immortalized CRC cell lines, Figure S3: sEVs characterization, Figure S4: Analysis of CD133 and CD147 cellular localization upon sEVs blocking, Figure S5: Flow cytometry of EVs bound to 4μm aldehyde/sulfate-latex beads. Figure S6–S13: Supplementary Blot.
