# Supplementary material for: CD147 Promotes Cell Small Extracellular Vesicles Release during Colon Cancer Stem Cells Differentiation and Triggers Cellular Changes in Recipient Cells
Source: Cancers (Basel). 2020 Jan 21;12(2):260. doi: 10.3390/cancers12020260 (PMC7072373; doi:10.3390/cancers12020260)
Supplement: Supplementary file 1 [file cancers-12-00260-s001.pdf]

Supplementary materials

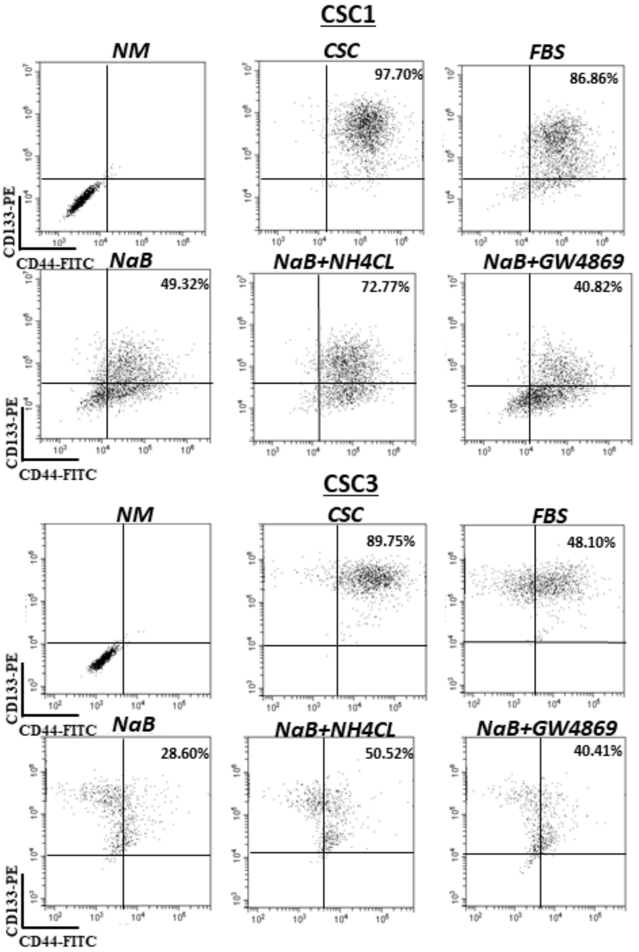

Figure S1. sEVs release is important for the differentiation of CR-CSCs. Analysis of CD133 and CD44 expression by flow cytometry in other CR-CSCs confirmed that differentiation decreased the double positive cells $CD133^+/CD44^+$ and that this effect was prevented by treatment with NH4CL or GW4869.

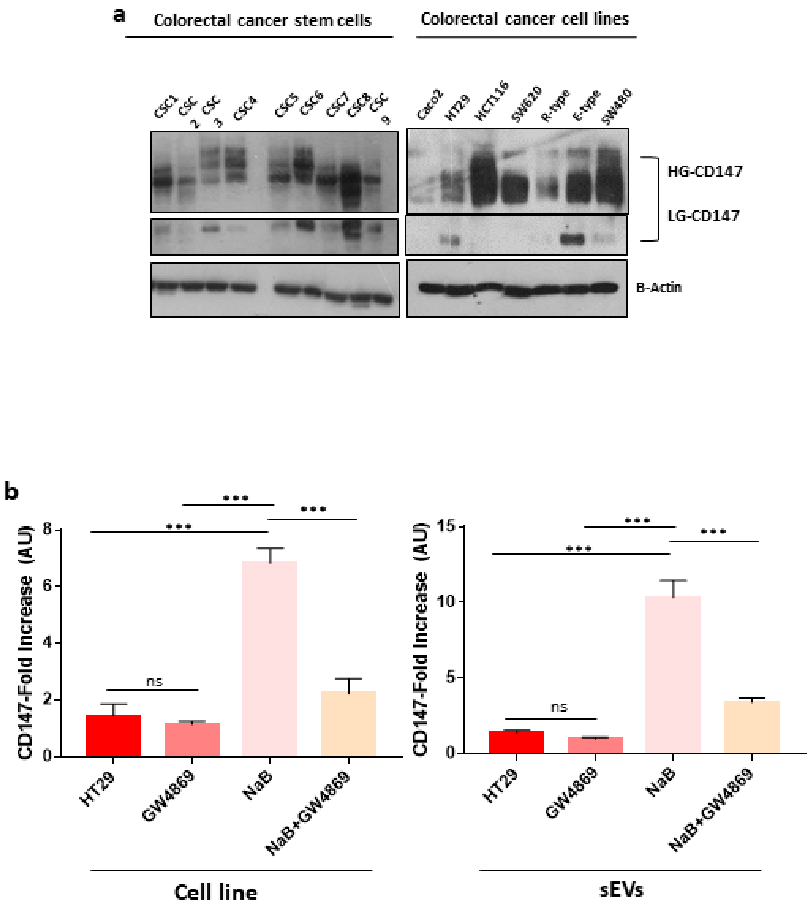

Figure S2. Evaluation of CD147 expression in CR-CSCs and immortalized CRC cell lines. (**a**) HG and LG indicate high and low-glycosylated CD147 weighting about 45–65 and 32kDa, respectively; (**b**) analysis of CD147 mRNA in HT29 cancer cells and in sEVs counterpart in basal and differentiated (NaB) conditions after treatment with an inhibitor of sEVs release (GW4869). ****, $p \leq 0.0001$.

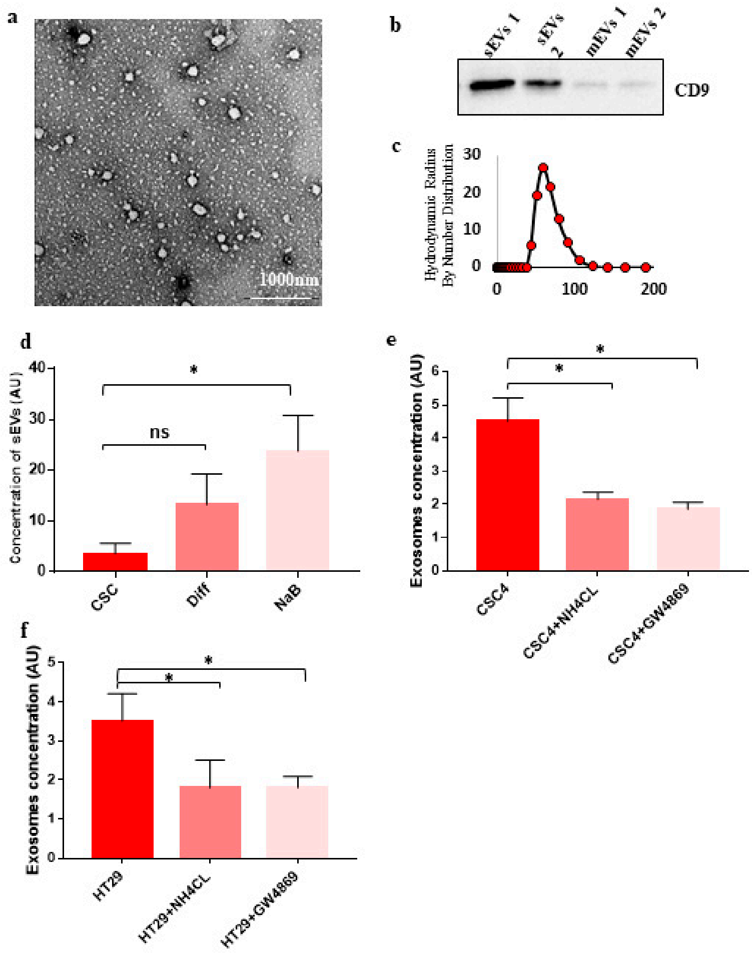

Figure S3. sEVs characterization. (**a**) Morphological analysis of sEVs by TEM; (**b**) expression of CD9 sEVs marker in sEVs and mEVs; (**c**) size analysis of sEVs by DLS confirming the correct dimension of isolated vesicles (sEVs< 200nm); (**d**) sEVs levels in the culture medium of CSC4 cells differentiated with 10% FBS, maintained in absence of growth factors (Diff.) and treated after seven days with 2mM NaB for 48 hours (NaB), as assessed by quantification of sEVs-extracted proteins (Bradford assay); (**e**) sEVs levels in the culture medium of CSC4 cells differentiated with NaB and treated with inhibitors of sEVs release (GW4869 and NH4CL); (**f**) sEVs levels in the culture medium of HT29 cells differentiated with NaB and treated with inhibitors of sEVs release (GW4869 and NH4CL). sEVs= extracellular vesicles <200nm; mEVs= extracellular vesicles > 200nm. * $p \leq 0.05$.

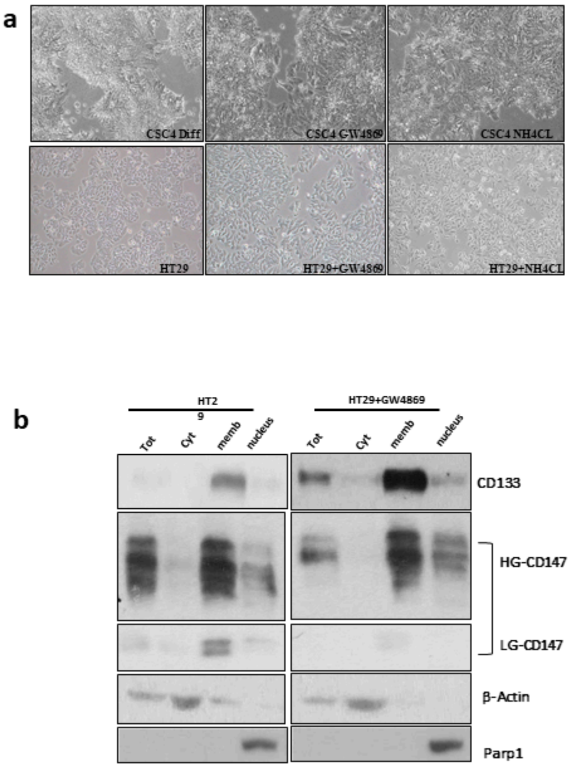

Figure S4. Analysis of CD133 and CD147 cellular localization upon sEVs blocking**.** (**a**) Morphology of CSC4 and HT29 cancer cells after the administration of sEVs inhibitors; (**b**) analysis of cellular localization of CD133 and CD147 upon blocking of sEV release.

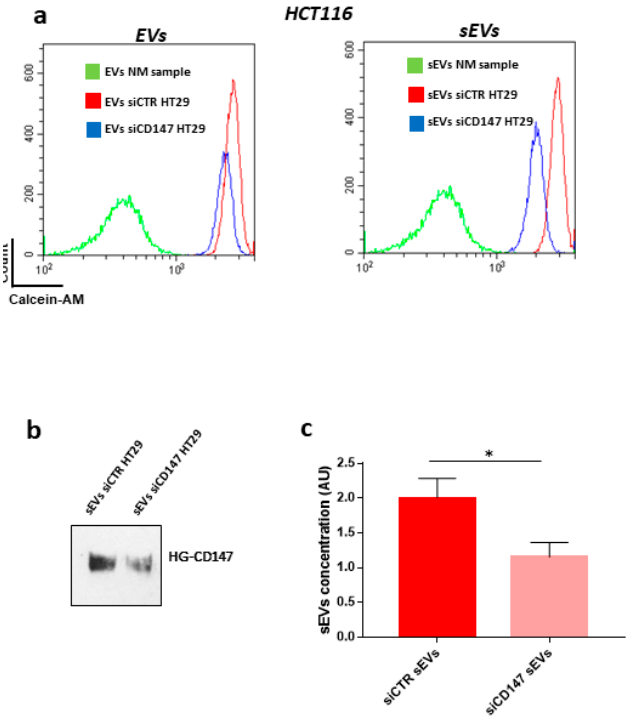

Figure S5. Flow cytometry of EVs bound to 4μm aldehyde/sulfate-latex beads. (**a**) Calcein-AM labelled EVs or sEVs were bound to 4μm aldehyde/sulfate-latex beads for 20 minutes at room temperature and then incubated with BSA 1.5% overnight on rotation. Bead-coupled EVs were pelleted by centrifugation at 2,000 g for 10 minutes, washed with 1 ml of BSA 0.5% and centrifuged again. The pellet was resuspended in PBS and acquired

by Cytoflex S (Beckman Coulter Indianapolis, IN 46268 United States) and analysed with CytExpert Software (Beckman Coulter Indianapolis, IN 46268 United States). Negative controls (NM) were obtained using beads coupled with unstained EVs or sEVs. (**b**) CD147 expression levels in sEVs released by HCT116 knockdown cells compared to mock transfected HCT116. (**c**) sEVs amount released by HCT116 transfected with siRNA for CD147 (siCD147) compared to HCT116 transfected with non-targeting control siRNA (SiCTR) tested by Bradford assay. * $p \leq 0.05$.

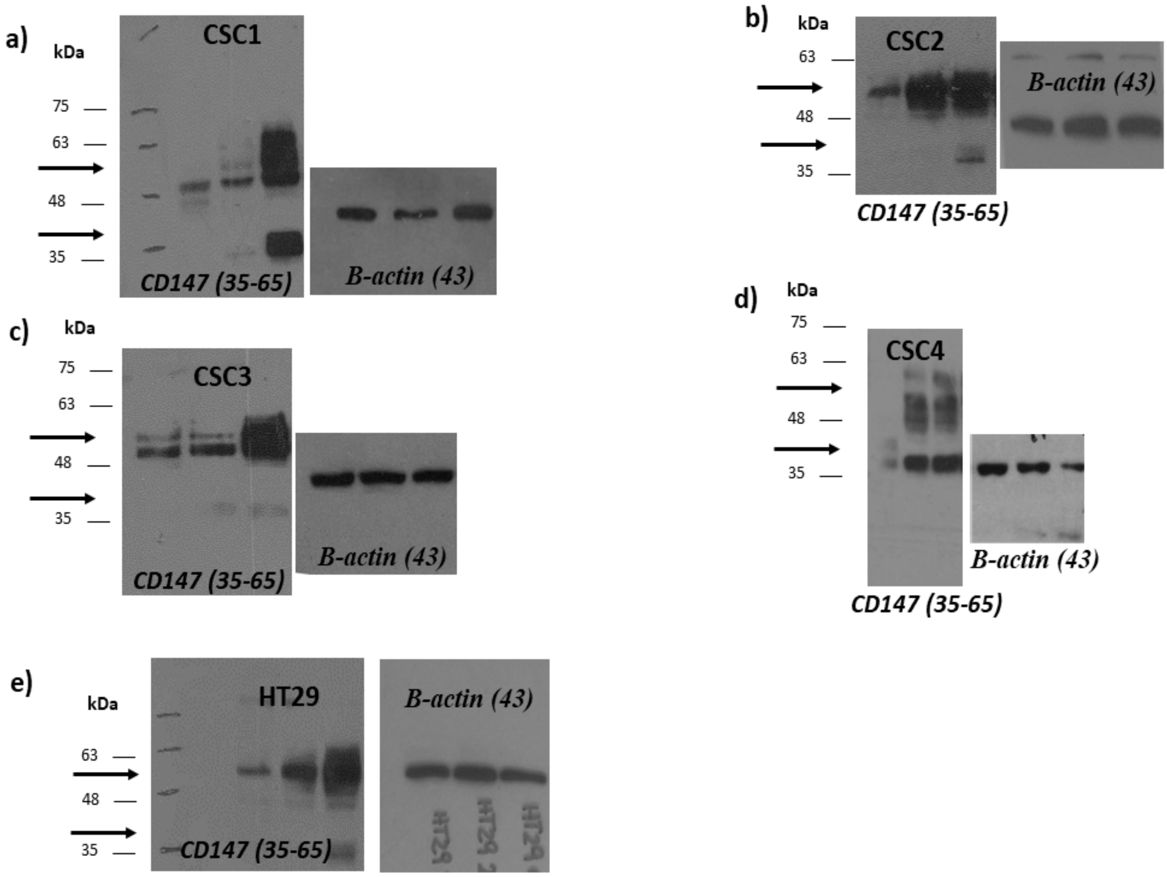

Figure S6. (**a**–**e**) CD147 expression in CSCs during differentiation.

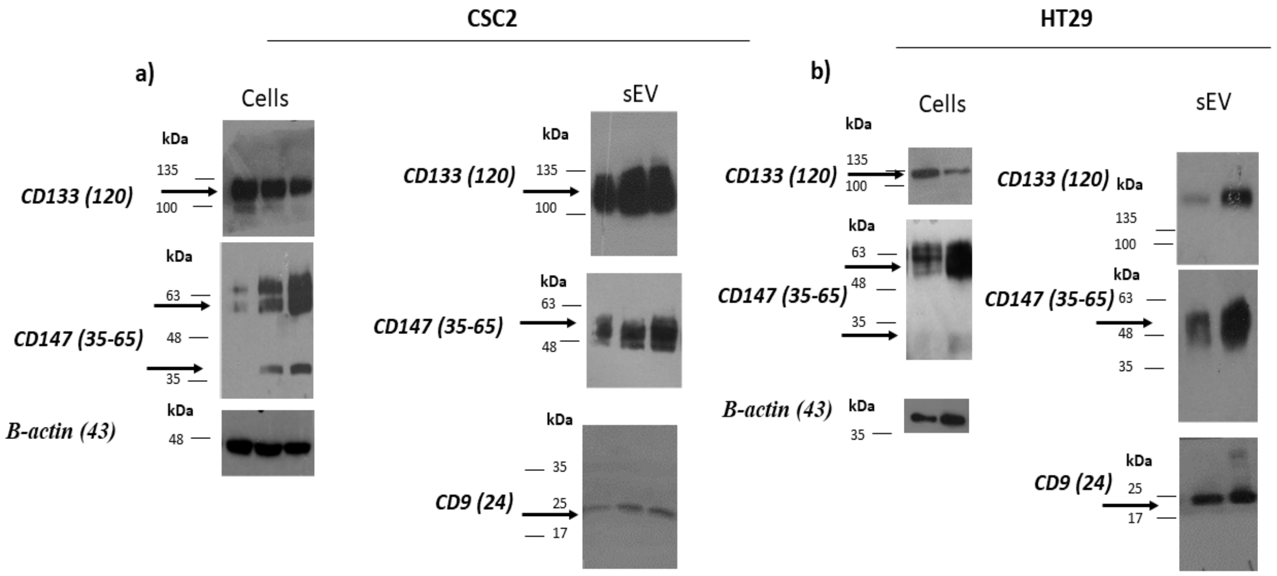

Figure S7. CD133 and CD147 expression in sEVs released by CR-CSC (**a**) and HT29 cells lines (**b**) undergoing differentiation.

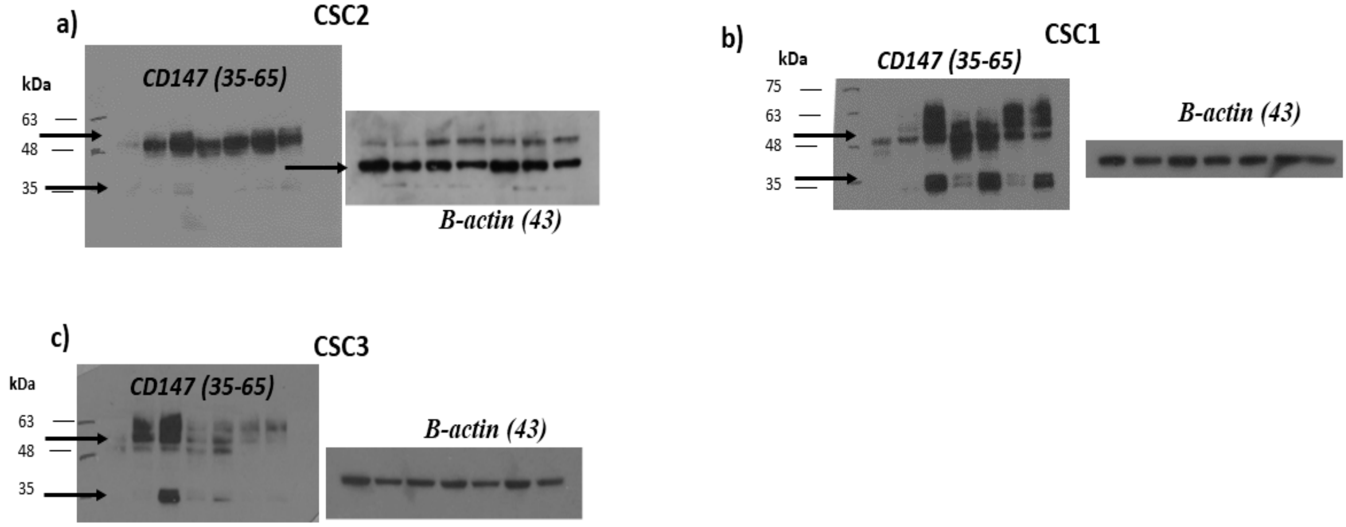

Figure S8. CD147 expression in CR-CSCs (**a**–**c**) treated with inhibitors of sEVs biogenesis during the differentiation.

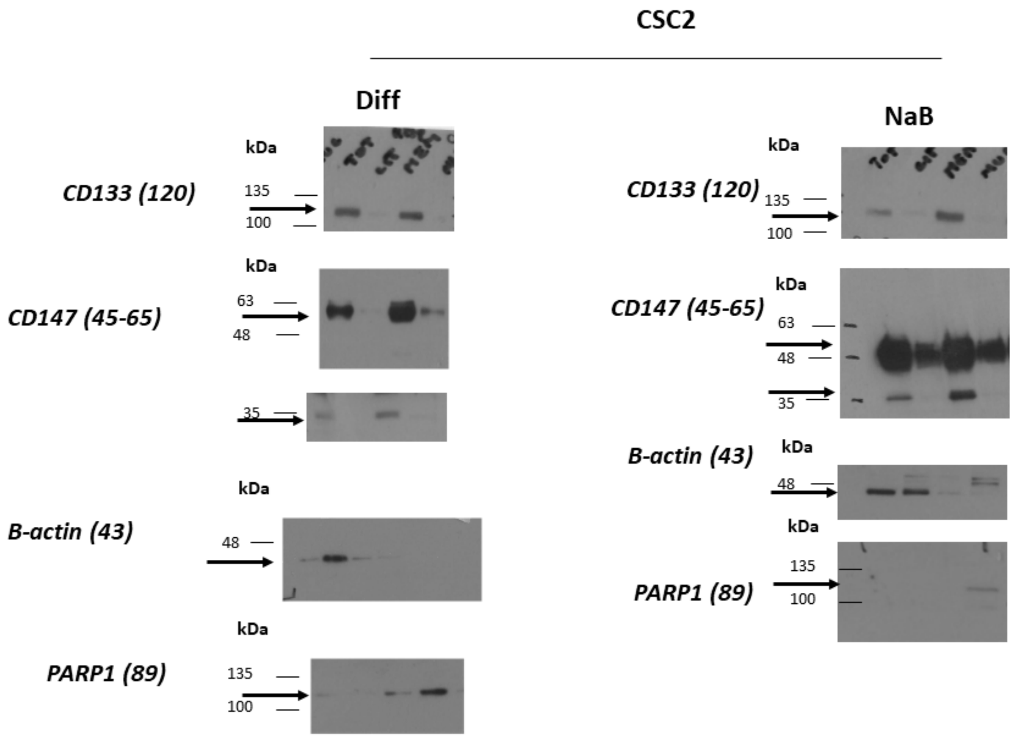

Figure S9. Characterization of CD147 subcellular localization in CR-CSCs.

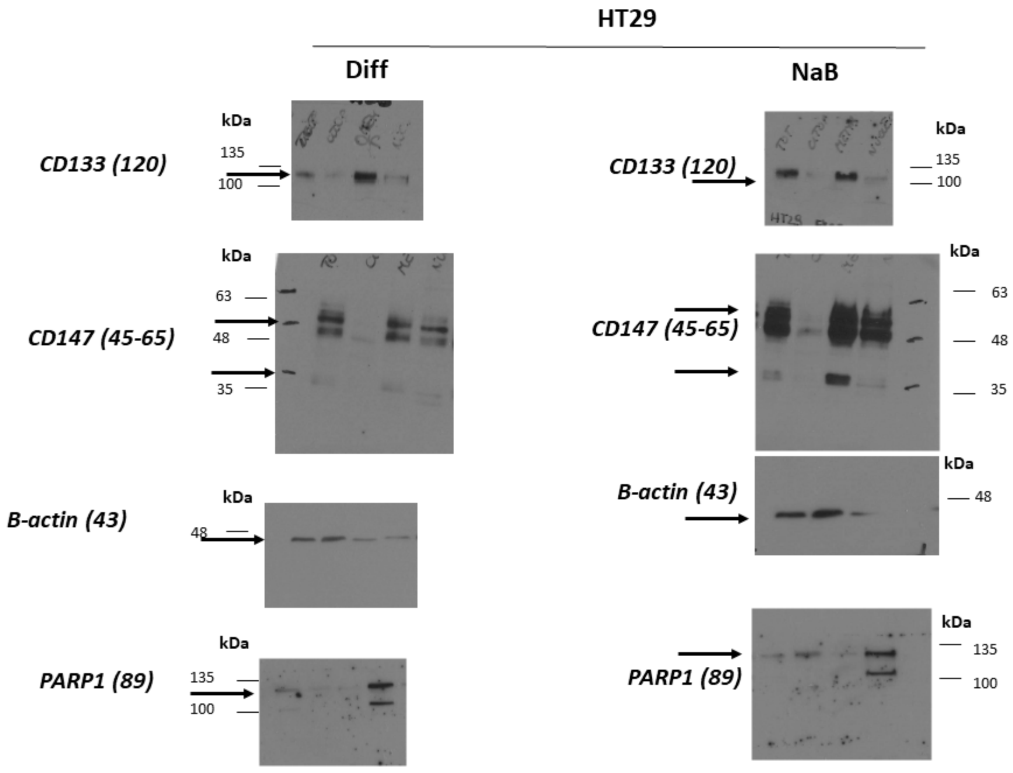

Figure S10. Characterization of CD147 subcellular localization in HT29 cell line.

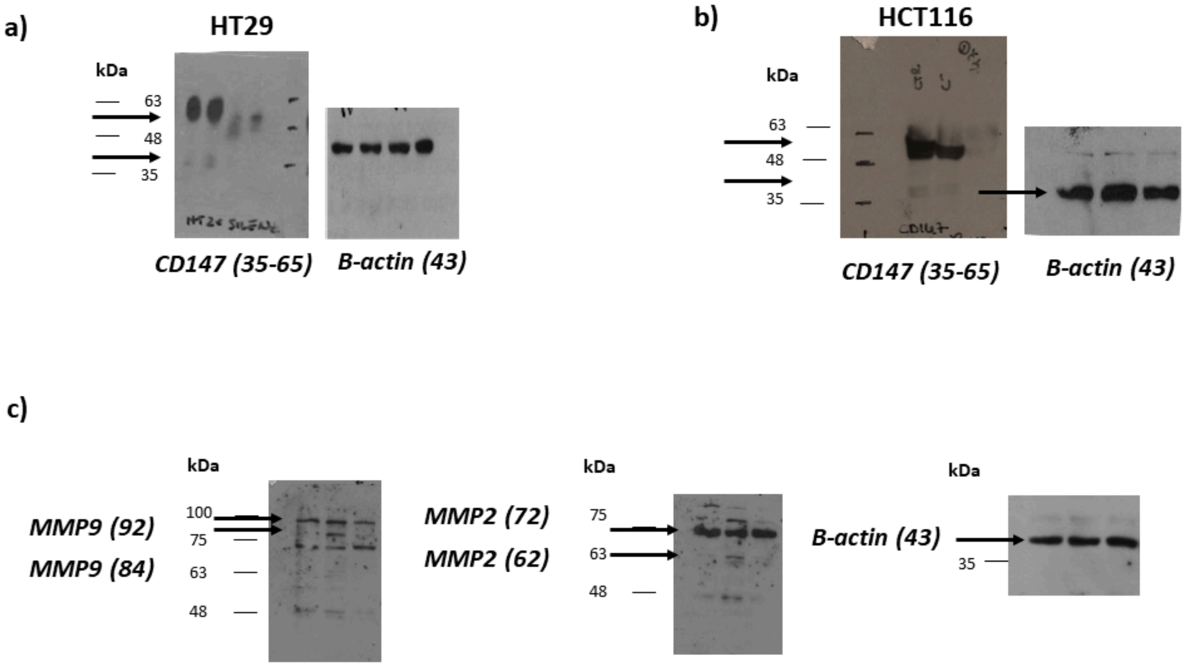

Figure S11. (**a**–**b**) CD147 expression after its knockdown in HT29 and HCT116 cancer cells; **(c)** blocking CD147 on sEVs surface prevented the activation of MMP2-MMP-9 expression.

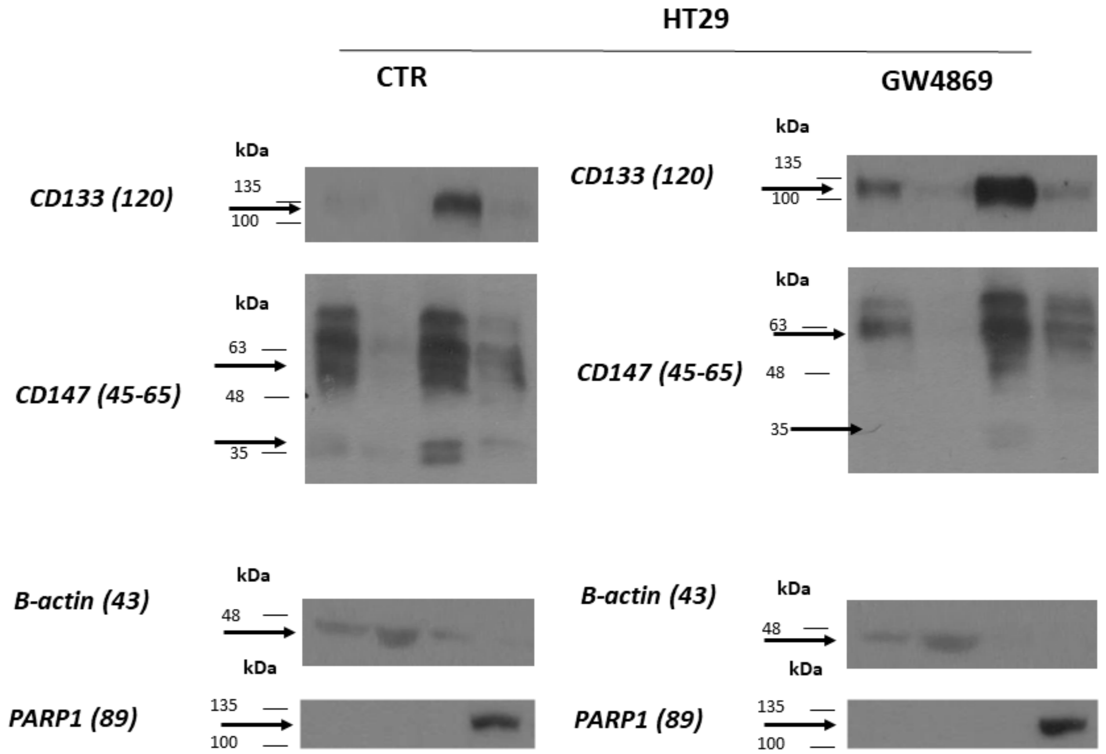

Figure S12. Analysis of CD133 and CD147 cellular localization upon sEVs blocking.

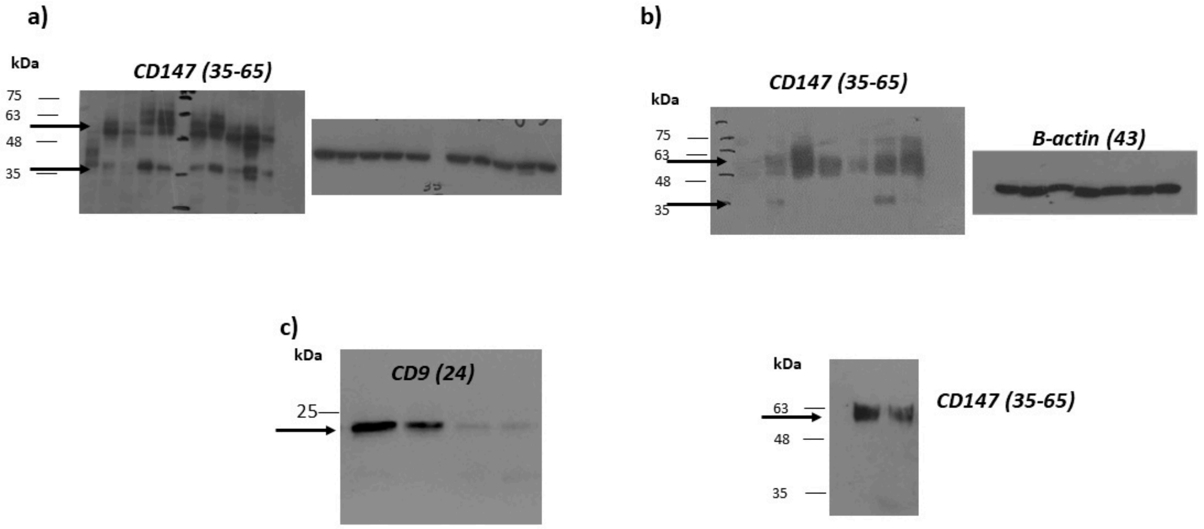

Figure S13. Evaluation of CD147 expression in CR-CSCs and immortalized CRC cell lines.
